# Rare-Earth Orthoferrites: Synthesis–Structure–Property Relationships, Functional Applications, and Future Perspectives

**DOI:** 10.3390/ijms27219770

**Published:** 2026-10-30

**Authors:** Mukhametkali Mataev, Moldir Abdraimova, Altynai Madiyarova, Karlygash Raissova, Marzhan Nurbekova, Bahadir Keskin

**Affiliations:** 1 Department of Chemistry, Faculty of Natural Sciences, Kazakh National Women’s Teacher Training University, Almaty 050000, Kazakhstan; mataev.m@qyzpu.edu.kz (M.M.); abdyraimova.m@qyzpu.edu.kz (M.A.); nurbekova.m@qyzpu.edu.kz (M.N.); 2 Department of Chemistry, Higher School of Natural Sciences and Information Technology, Sarsen Amanzholov East Kazakhstan University, Ust-Kamenogorsk 070000, Kazakhstan; 3 Department of Chemistry, Faculty of Arts & Science, Yildiz Technical University, TR34220 Istanbul, Turkey; bahadirkeskin@gmail.com

**Keywords:** rare-earth orthoferrites, RFeO_3_, perovskite oxides, synthesis methods, cation substitution, oxygen vacancies, structure–property relationships, magnetic properties, sensing materials, photocatalysis, electrocatalysis

## Abstract

Rare-earth-based RFeO_3_ orthoferrites are multifunctional materials distinguished by their structural flexibility, high chemical and thermal stability, and unique combination of magnetic, electrical, dielectric, optical, sensing, and catalytic properties. This review critically systematizes recent advances in the synthesis of RFeO_3_ orthoferrites, the development of their structural and morphological characteristics, and the targeted control of their functional properties. The effects of solid-state reaction, optical floating-zone growth, sol–gel processing, sol–gel auto-combustion, coprecipitation, hydrothermal synthesis, and microwave-assisted synthesis on phase purity, crystallinity, particle size, morphology, and defect structure are compared. The influence of A- and B-site cation substitution, oxygen vacancies, FeO_6_ octahedral tilting, and microstrain on charge transport, magnetic exchange interactions, and electronic structure is examined. The capabilities of X-ray diffraction (XRD), X-ray photoelectron spectroscopy (XPS), Raman and Fourier-transform infrared (FTIR) spectroscopy, electron microscopy, vibrating-sample magnetometry (VSM), linear sweep voltammetry (LSV), and electrochemical impedance spectroscopy (EIS) for elucidating structure–property relationships are assessed. The compiled evidence demonstrates that synthesis conditions and chemical modification produce interrelated changes in the phase composition, grain size, band-gap energy, dielectric and magnetic responses, and sensing and catalytic activity of orthoferrites. The application potential of RFeO_3_-based materials in magnetic refrigeration, spintronics, supercapacitors, gas sensors, solid oxide fuel cells, magnetic memory, capacitors, photocatalysis, and electrocatalysis is evaluated. The analysis identifies the systematic elucidation of the “synthesis–structure–morphology–property–function” relationship, quantitative characterization of defect chemistry, standardization of measurement protocols, and evaluation of long-term operational stability as key priorities for future research.

## 1. Introduction

The rapid development of nanotechnology as an interdisciplinary field has substantially accelerated scientific and technological progress in engineering, environmental remediation, optics, electrical engineering, chemistry, biology, medicine, materials science, and photocatalysis [1,2,3,4,5]. The distinctive characteristics of nanomaterials arise from their dimensions in the 1–100 nm range, high specific surface area, tunable size and shape, unique density of energy states, short intraparticle diffusion lengths, high surface free energy, and unusual physicochemical properties [2,3,4,5,6,7,8,9]. One of the principal advantages of nanotechnology-based approaches is the ability to precisely tailor the dimensional and morphological parameters of materials [6].

Among the diverse classes of nanomaterials, magnetic nanoparticles have attracted considerable research interest because their physicochemical and magnetic characteristics differ markedly from those of their bulk counterparts [10,11]. Their functional versatility is reflected in applications including magnetic resonance imaging, targeted drug-delivery systems, cell labeling, damaged-tissue repair, enzyme and protein immobilization, and antioxidant and antibacterial materials [10,11,12,13]. Magnetic nanoparticles also offer substantial practical potential in technologies for removing environmental pollutants. Their facile separation under an external magnetic field, reusability, high magnetic susceptibility, amenability to surface functionalization, large specific surface area, and high reactivity enhance their value in environmental remediation [10,11,12,13,14]. Their magnetic behavior, however, is directly governed by particle size, geometry, crystallinity, and structural defects [10].

**Figure 1:**
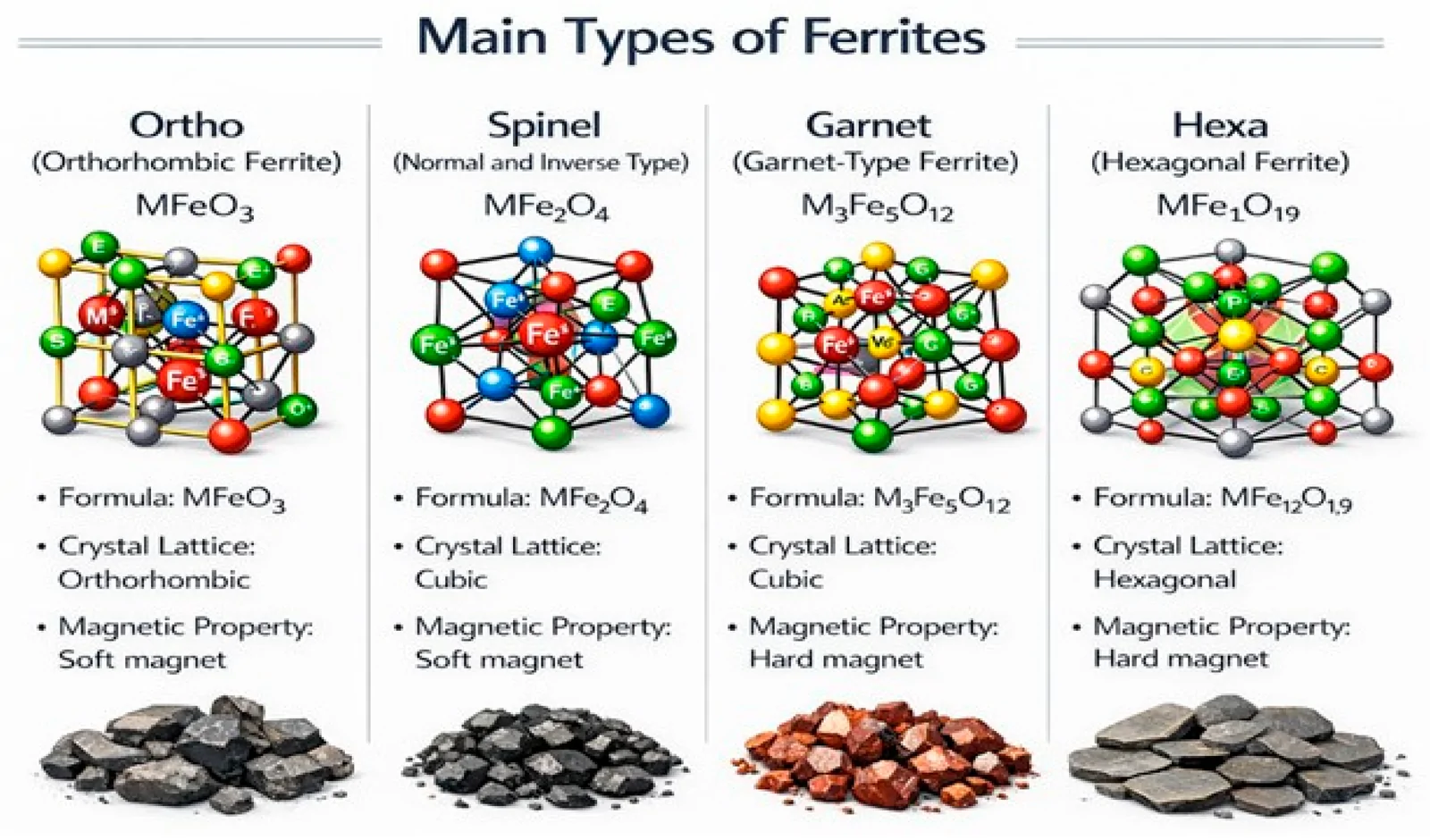
Four classes of ferrites: garnet-type, orthoferrite, hexagonal, and spinel ferrites [17].

Ferrites constitute an important class of iron-based magnetic oxide materials. Their magnetic behavior is determined by the relative orientation and incomplete compensation of magnetic moments within the crystal lattice. The physicochemical and magnetic properties of nanoscale ferrites depend on their chemical composition, particle size and morphology, crystal structure, and interactions with the surrounding environment. According to their crystal structure, ferrites are classified as spinel, perovskite or orthoferrite, garnet, and magnetoplumbite (hexagonal) ferrites. In these structures, the distribution of oxygen ions and metal cations over different crystallographic sites directly governs the structural, magnetic, and functional characteristics of the materials [15,16]. As shown in Figure 1, ferrites are classified according to their crystal structure and chemical composition into orthorhombic orthoferrites (MFeO_3_), spinel ferrites (MFe_2_O_4_), garnet-type ferrites (M_3_Fe_5_O_12_), and hexagonal ferrites (MFe_12_O_19_).

Among ferritic materials, perovskite oxides with the general formula ABO_3_ are notable for their structural flexibility and broad spectrum of functional properties, including ferroelectricity, magnetism, superconductivity, dielectric response, catalytic activity, and electrical conductivity [18,19]. The strong coupling among charge, spin, orbital, and lattice degrees of freedom makes perovskite oxides promising functional materials for sensors, actuators, spintronic devices, energy-conversion systems, photocatalysis, and environmental remediation technologies [20]. In the perovskite structure, the A site is predominantly occupied by rare-earth or alkaline-earth cations, whereas the B site accommodates transition-metal ions such as Cr, Mn, Fe, and Co. This compositional flexibility enables the structural, physicochemical, and functional characteristics of the material to be tuned over a wide range through targeted cation substitution at the A and B sites [21,22,23,24].

**Figure 2:**
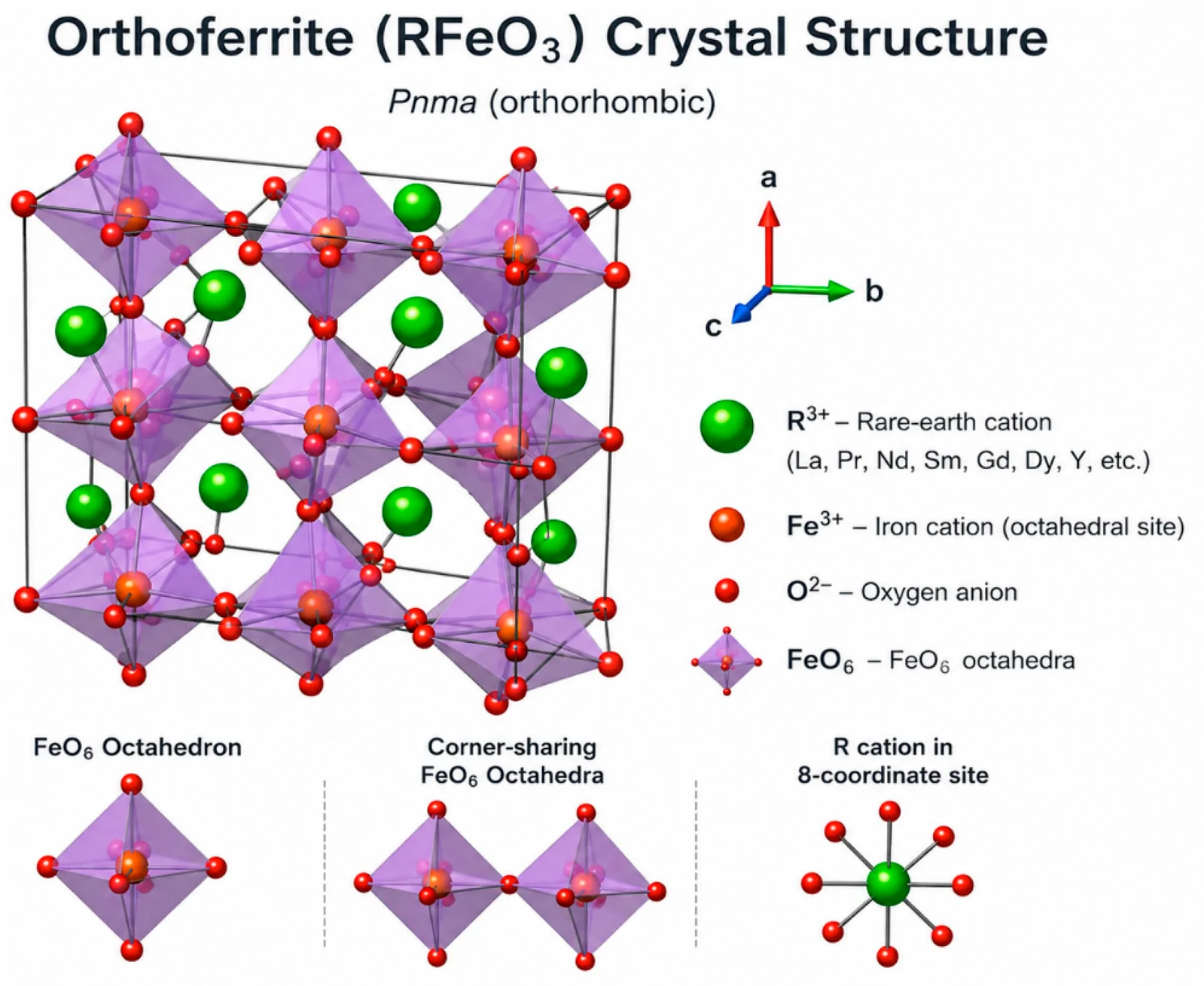
Crystal structure of an orthoferrite.

Rare-earth-based RFeO_3_ orthoferrites, where R denotes a rare-earth ion or Y, form a particularly important family of perovskite oxides. In addition to high thermal and chemical stability, they exhibit a distinctive combination of magnetic, optical, electrical, dielectric, and catalytic properties [25]. RFeO_3_ orthoferrites generally crystallize in a distorted orthorhombic perovskite structure belonging to the Pnma space group. They exhibit antiferromagnetic ordering accompanied by weak ferromagnetism originating from spin canting induced by the Dzyaloshinskii–Moriya interaction between Fe^3+^ ions [26]. Charge-transfer transitions within the Fe–O framework enable efficient absorption of visible light, thereby increasing the potential of orthoferrites for photocatalysis, spintronics, magnetic sensors, magnetoelectric devices, and environmental remediation technologies [27,28]. Understanding these functional properties requires consideration of the crystal structure: as shown in Figure 2, RFeO_3_ orthoferrites adopt an orthorhombic perovskite structure with the Pnma space group, consisting of corner-sharing FeO_6_ octahedra. Distortions and tilting of these octahedra modify the Fe–O–Fe bond geometry, influencing their magnetic and electronic properties.

Interest in the antiferromagnetic nature and multifunctional properties of RFeO_3_ orthoferrites has increased substantially in recent years. Their potential use in high-speed spintronic devices [29,30,31], supercapacitor electrodes [32], gas and humidity sensors [33,34,35,36], photocatalysts [37], optoelectronic components [38,39], and other functional systems is being actively explored. Furthermore, multifunctional phenomena such as the piezocatalytic activity of ABO_3_-type perovskites further broaden their range of practical applications [40].

Among sensing applications, the development of advanced functional materials for humidity detection is of particular importance. A number of currently available commercial humidity sensors suffer from insufficient long-term stability, hysteresis, slow response, and high manufacturing costs [41,42]. Accordingly, the development of new sensing materials that combine high sensitivity, rapid response, stability, and cost-effective fabrication remains an important research objective. The structural flexibility of ABO_3_-type perovskites, the wide tunability of their electrical characteristics, and their high chemical stability make them promising platforms for the development of sensing devices [43,44].

The functional properties of RFeO_3_ systems are governed not only by the identity of the rare-earth cation but also by the degree of lattice distortion, FeO_6_ octahedral tilting, defect concentration, oxygen-vacancy content, particle size, and microstructural state. Among rare-earth orthoferrites, HoFeO_3_ is of particular interest because of the large magnetic moment of Ho^3+^ ions, strong spin–orbit coupling, and pronounced magnetic anisotropy [45]. Interactions between the Ho^3+^ and Fe^3+^ magnetic sublattices influence spin dynamics, magnetic exchange, and the distortion of FeO_6_ octahedra. These structural changes affect charge transport, phonon dynamics, magnetic ordering, and surface catalytic activity. The relatively narrow band gap, visible-light absorption capability, chemical stability, and multifunctional nature of HoFeO_3_ make it a promising material for photocatalytic and electronic devices [46].

An important functional feature of orthoferrites is their negative temperature coefficient of resistance (NTCR). In such materials, electrical resistance decreases with increasing temperature as a result of thermally activated charge transport [47]. This behavior is commonly attributed to small-polaron hopping between localized Fe^2+^/Fe^3+^ states and depends on crystallinity, oxygen-vacancy concentration, grain-boundary resistance, and microstructural state. These characteristics support the use of orthoferrite materials in temperature sensors, thermal switches, and electronic control components.

Chemical modification plays a central role in the targeted tailoring of RFeO_3_ orthoferrite properties. In addition to pristine compounds, substitution at the R and/or Fe sites and co-doping strategies have been widely investigated. Cation substitution affects lattice strain, FeO_6_ octahedral distortion, oxygen-vacancy concentration, particle size, charge-transport processes, and magnetic interactions, thereby enabling controlled adjustment of functional properties. To enhance photocatalytic performance, doping with transition-metal or rare-earth ions is used to modify the electronic band structure, extend visible-light absorption, and suppress electron–hole recombination. The formation of heterostructures or composites with TiO_2_, g-C_3_N_4_, ZnO, and graphene-based materials can additionally improve interfacial charge transfer and accelerate photocatalytic reaction kinetics. Control of oxygen vacancies, morphological engineering, surface modification, and optimization of thermal-treatment conditions are likewise important strategies for regulating the generation of reactive oxygen species and surface adsorption processes [48].

In addition to chemical composition, the synthesis route and subsequent thermal-treatment conditions are key determinants of the structure and properties of orthoferrites. Solid-state reaction, sol–gel processing, combustion synthesis, coprecipitation, hydrothermal synthesis, and other preparation methods exert distinct effects on phase purity, crystallinity, chemical homogeneity, particle size and morphology, and defect concentration. In particular, the calcination temperature influences crystallinity, lattice strain, oxygen-vacancy content, grain growth, grain-boundary characteristics, and surface reactivity. Optimization of synthesis parameters is therefore an essential means of tailoring the electrical, dielectric, magnetic, optical, sensing, and catalytic properties of orthoferrites.

Despite the growing body of research on RFeO_3_ orthoferrites, several important scientific issues have yet to be comprehensively systematized. A substantial proportion of published studies focus on individual synthesis routes, the effect of a particular dopant, or a single functional property. The coupled effects of synthesis conditions, doping chemistry, microstructural evolution, lattice distortion, defect structure, and oxygen vacancies on the magnetic, dielectric, electrical, optical, sensing, and catalytic behavior of RFeO_3_ materials have not yet been sufficiently integrated within a unified framework. In particular, establishing the “synthesis–structure–morphology–property–function” relationship and comparatively evaluating findings across different studies are essential for the rational optimization of the composition and structure of multifunctional orthoferrite materials.

Against this background, this review aims to provide a systematic analysis of advances in the synthesis of rare-earth-based RFeO_3_ orthoferrites, the development of their structural and morphological characteristics, and the control of their functional properties. Particular emphasis is placed on comparing the effects of different synthesis routes and thermal-treatment parameters on phase composition, crystallinity, particle size, morphology, and defect structure; elucidating how cation substitution and doping at the A and B sites affect structural changes, charge transport, and magnetic interactions; and assessing the relationships between structural and microstructural features and magnetic, dielectric, electrical, optical, sensing, and catalytic properties. The review also examines the application prospects of RFeO_3_-based materials in sensors, spintronic and magnetoelectric devices, energy-storage and conversion systems, photocatalysis, and environmental remediation technologies, and identifies the principal scientific and technological challenges and priorities for future research in this field.

## 2. Synthesis Methods for Rare-Earth Orthoferrites

**Figure 3:**
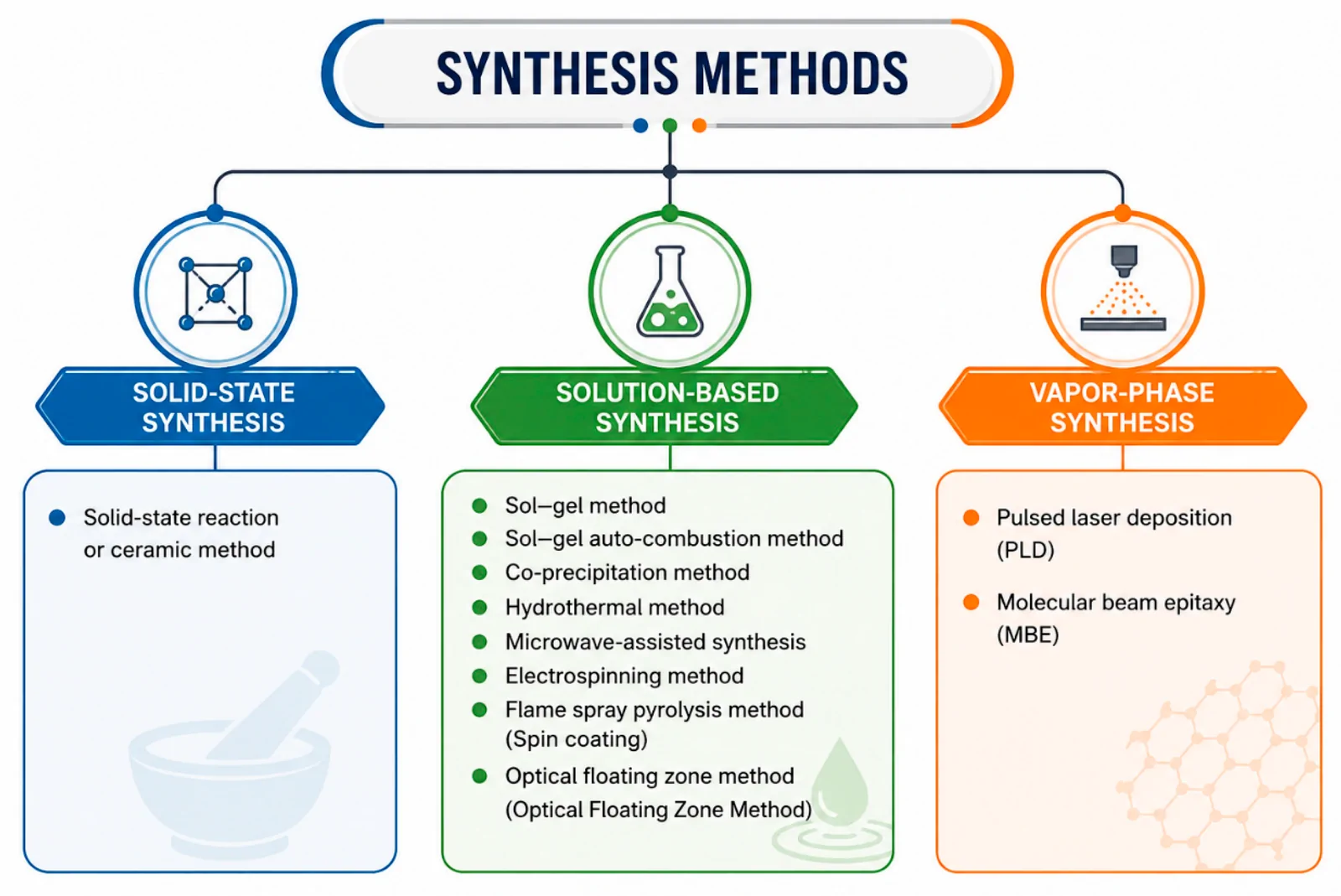
Classification of synthesis methods for orthoferrite materials.

Because the structure and functional characteristics of RFeO_3_ orthoferrites are directly dependent on the synthesis route, method selection should be governed not only by technological convenience but also by the targeted phase purity, particle size, morphology, and crystallinity. Depending on the physical state of the starting reagents and the nature of the reaction pathway, orthoferrite preparation methods can be classified into solid-phase, liquid-phase, and gas-phase routes; an overview of this classification is presented in Figure 3.

RFeO_3_ systems have been prepared using solid-state reactions [49,50,51,52,53,54,55,56,57,58,59,60], the optical floating-zone method [61,62,63,64,65,66], sol–gel processing [67,68,69,70,71,72,73,74,75], sol–gel auto-combustion [76,77,78,79], coprecipitation [80,81,82,83,84,85], hydrothermal synthesis [86,87,88,89,90], microwave-assisted hydrothermal synthesis [91,92,93], and specialized techniques for fabricating thin films and one-dimensional structures. These methods should not be regarded as interchangeable routes, but rather as technological strategies designed to achieve distinct structural outcomes.

The principal advantages of solid-state synthesis are its technological simplicity and scalability, whereas its main limitations are particle growth, agglomeration, and the risk of secondary-phase formation caused by high temperatures and prolonged diffusion-controlled processing. Accordingly, this method is better suited to the production of bulk ceramics than to the precise control of nanoscale dimensions and morphology.

Wet-chemical methods enable precursor mixing at the solution level, thereby facilitating phase formation at relatively low temperatures and providing greater flexibility in controlling particle size and morphology [94]. Their use, however, does not inherently guarantee superior material quality: variations in pH, concentration, gelation, precipitation, and subsequent calcination conditions can substantially alter the resulting structure.

These differences indicate that a synthesis route should be evaluated not according to the notion of a universally “most efficient method,” but in terms of its compatibility with the required material form and intended application. The following sections compare the structural outcomes, degree of process control, and technological limitations of each method from this perspective.

### 2.1. Solid-State Reaction Method

**Figure 4:**
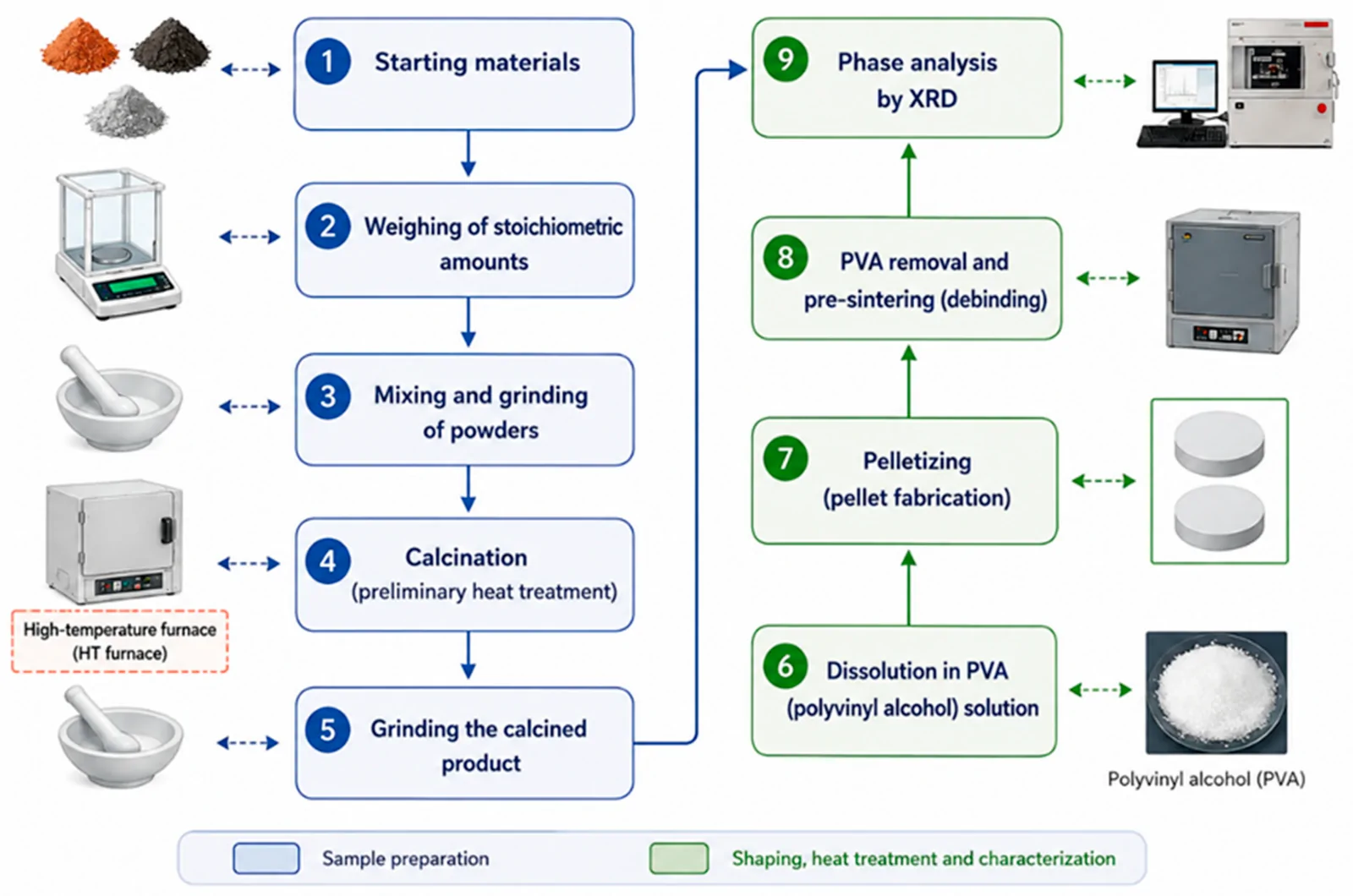
Process flow diagram for the synthesis of orthoferrite materials via the solid-state reaction (ceramic method).

The solid-state reaction is a conventional ceramic route for preparing perovskite materials (Figure 4). Based on oxides, carbonates, nitrates, and other solid precursors, the method is widely used because of reagent availability, relatively low cost, and technological simplicity. Nevertheless, any assessment of its effectiveness must balance these advantages against the requirements for high-temperature treatment and the associated diffusion limitations.

Slow diffusion between solid components prolongs the reaction and typically requires calcination or sintering temperatures of 900–1500 °C for orthoferrites [49,51,54,57]. Although such conditions promote phase formation, they hinder precise control over particle size and specific surface area, thereby limiting the ability to obtain nanoscale products.

The reported variation in particle size from 10–15 nm [95 to 5.2–5.6 μm [55], with intermediate values of 27–88 nm [52,59,60,95,96] and <50 nm [97], demonstrates that the solid-state method does not produce a single consistent morphological outcome. This broad dispersion indicates that particle size depends more strongly on precursor chemistry and thermal conditions than on the nominal method itself; consequently, results from different studies cannot be meaningfully compared without considering their synthesis parameters.

Phase purity likewise does not improve monotonically with increasing temperature. Xiong et al. [98] showed that Er_3_Fe_5_O_12_, rather than the target phase, formed in the ErFeO_3_ system at 1250 and 1500 °C, whereas single-phase ErFeO_3_ was obtained between 1300 and 1450 °C; 1300 °C was identified as optimal for producing high-quality polycrystalline ceramics. Thus, high temperature alone is insufficient in solid-state synthesis, and identifying a narrow processing window is essential for maintaining stoichiometric homogeneity and suppressing secondary phases [98,99].

### 2.2. Optical Floating-Zone Method

The principal value of the optical floating-zone method lies in its ability to grow RFeO_3_ single crystals with low defect densities without the use of a crucible or flux. Its functional purpose therefore differs from that of methods designed to produce polycrystalline powders: rather than minimizing particle size, the primary objective is to maximize crystal quality.

Alongside its capacity to yield highly crystalline materials, the method is constrained by pronounced process sensitivity. The formation of dislocations, cracks, and gas bubbles depends on the stability of the molten zone, the quality and rotation rates of the feed and seed rods, the temperature field, atmosphere, composition, and growth rate [62,63,100,101]. Precise parameter control is therefore not an auxiliary requirement but the principal determinant of single-crystal quality.

Wang et al. [100] demonstrated the decisive role of crystal growth rate in NdFeO_3_, whereas Sivakumar et al. [101] grew LaFeO_3_ and Sr-doped LaFeO_3_ single crystals and investigated their dielectric and magnetic properties. These findings confirm that the key advantage of the optical floating-zone method is its ability to produce high-quality single crystals, although its effectiveness depends on rigorous multivariable process control.

### 2.3. Sol–Gel Method

For multicomponent oxides such as RFeO_3_, the sol–gel method improves chemical homogeneity by mixing precursors at the solution level and enables phase formation at lower temperatures than solid-state synthesis [102,103]. The process sequence comprises sol formation, gelation, drying, and thermal treatment. Thus, although the method affords a high degree of control, the final structure remains sensitive to the conditions employed at each stage.

The principal advantage of sol–gel processing is the ability to tune particle size and structure through precursor composition and thermal conditions. Gosavi et al. [104] found that sol–gel-derived LaFeO_3_ exhibited higher phase purity, a porous structure, and a specific surface area of 16.5 m^2^ g^−1^ compared with samples prepared by coprecipitation and combustion methods. This comparison does not establish a universal superiority of the method; rather, it demonstrates its suitability for specific structural requirements, particularly high surface area and chemical homogeneity.

Particle sizes of approximately 65 nm have been reported for sol–gel-derived SmFeO_3_ [105], 40–80 nm for LnFeO_3_ (Ln = La, Sm) [106], and 20–252 nm for doped systems [68,70,107,108]. This broad range indicates that doping and subsequent heat treatment influence particle size at least as strongly as the synthesis route itself. Consequently, describing sol–gel processing merely as a “method for producing nanoscale materials” is insufficient.

### 2.4. Sol–Gel Auto-Combustion Method

Sol–gel auto-combustion combines the chemical homogeneity of a sol with rapid phase formation driven by an exothermic self-combustion reaction [109,110,111]. The use of nitrates or acetates together with fuels such as glycine, urea, or citric acid makes the method technologically straightforward and relatively economical; however, the outcome is strongly dependent on fuel type and combustion conditions.

The method can provide high chemical homogeneity, phase purity, crystallinity, and specific surface area [76,112]. Nevertheless, grain growth and enhanced agglomeration during subsequent heat treatment may diminish the initial nanostructural advantages. The combustion stage and post-synthesis thermal treatment must therefore be optimized as an integrated processing system.

**Figure 5:**
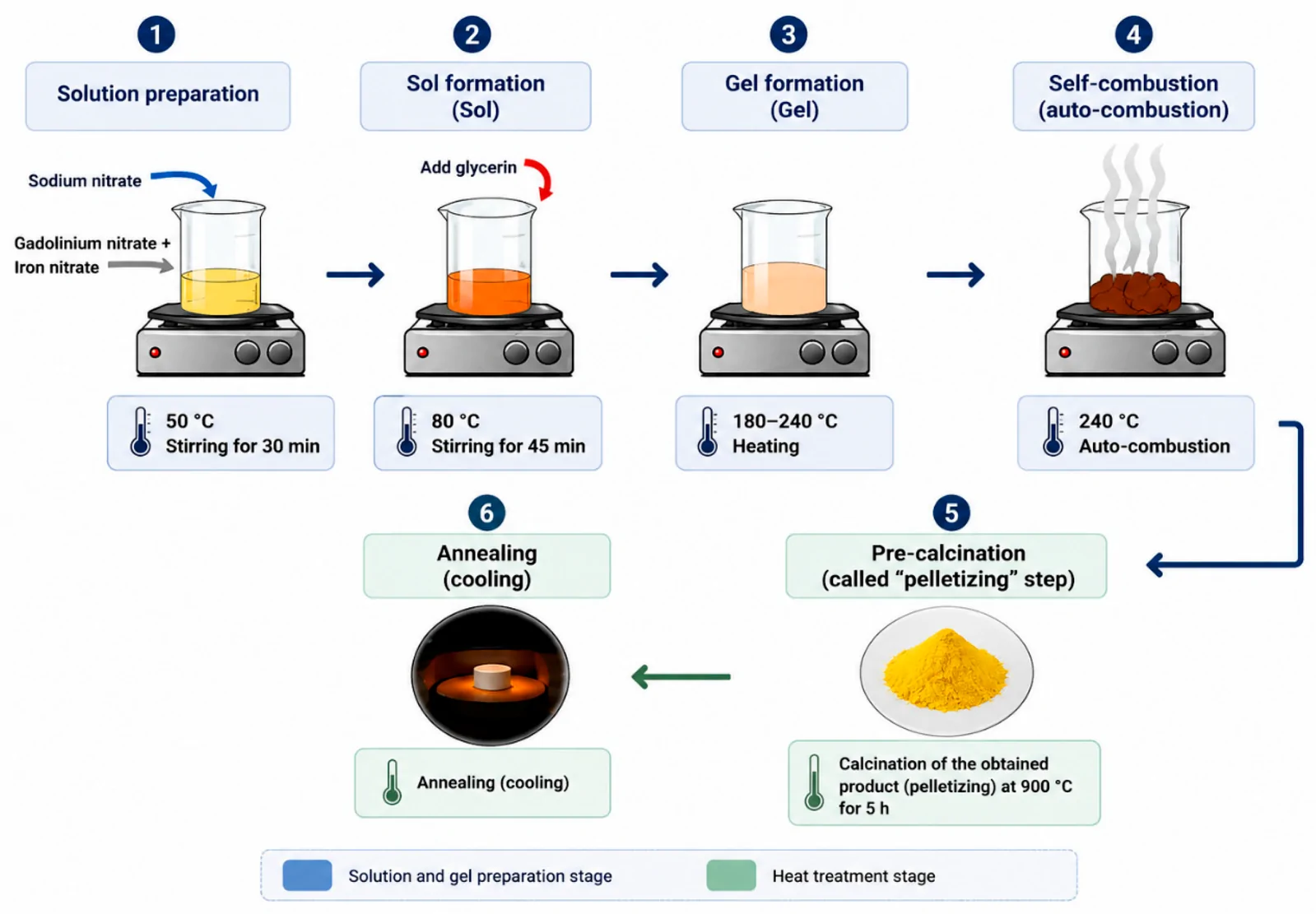
Process flow diagram for the synthesis of Gd_1−x_Na_x_FeO_3_ (x = 0, 0.05, 0.1, and 0.2) orthoferrites by the sol–gel auto-combustion method [101].

Reported particle or crystallite sizes range from 15–25 nm [113] and 24–50 nm [102,111,112,114] to 100–150 nm [79,115] and 173–365 nm [116]. This wide range demonstrates that auto-combustion does not inherently guarantee fine particles and highlights the decisive role of subsequent calcination. The Gd_1−x_Na_x_FeO_3_ (x = 0–0.2) system investigated by Krishnan et al. [112] illustrates the adaptability of this method to compositional modification (Figure 5).

### 2.5. Coprecipitation Method

In coprecipitation, metal cations are simultaneously precipitated from a single solution as hydroxides, carbonates, oxalates, or citrates and subsequently calcined [117,118,119]. The principal advantage of the method is the high degree of component mixing in solution, whereas its main limitation is the need to precisely match solubility characteristics and solution conditions to achieve simultaneous and homogeneous precipitation of all cations.

Nguyen et al. [80] obtained HoFeO_3_ crystallites of 25–30 nm, whereas Gupta et al. [120] showed that SmFeO_3_ prepared by low-temperature coprecipitation exhibited greater phase and morphological homogeneity than its solid-state-derived counterpart. These findings indicate that the advantages of coprecipitation arise not from low temperature alone, but from the homogeneous distribution of precursors at the solution level.

At the same time, the variation in reported sizes from 18.2–77 nm [83,84,85,117] to 0.2–2 μm [121] demonstrates that morphology is not determined solely by the precipitation process. The formation of spindle-like structures in addition to spherical and pseudospherical particles [85,112,121] confirms that pH, concentration, temperature, and subsequent calcination are equally important determinants of morphology.

Thus, although coprecipitation is effective for producing fine, chemically homogeneous powders, it requires precise control of pH, concentration, temperature, and stirring rate [81,121]. The need for prolonged calcination [118] partially offsets its advantages in terms of energy and time efficiency.

### 2.6. Hydrothermal/Solvothermal Method

Hydrothermal/solvothermal synthesis is conducted in an autoclave under controlled temperature and pressure, thereby reducing either the need for subsequent thermal treatment or the required treatment temperature. Its principal advantage is the ability to produce highly phase-pure particles with controlled morphology, whereas its main technological limitations are the requirement for high-pressure equipment and stringent safety measures.

Reported hydrothermal product sizes range from 30–70 nm [86] and 31–39 nm [87] to 260–427 nm [89]. Although these findings demonstrate the potential of the method to produce narrow size distributions, they also show that the absolute particle size depends on temperature, pH, precursor concentration, and reaction time [103,122].

Nakhaei et al. [89] reported higher magnetization for hydrothermally synthesized orthoferrites than for sol–gel-derived samples. Such a difference cannot, however, be attributed solely to the name of the synthesis method, because morphology, crystallinity, and particle size also vary concurrently. The ability to obtain nanospheres, nanoflowers, nanoplates, nanorods, and core–shell structures by varying hydrothermal parameters [102,122,123] demonstrates that the principal strength of this method is its morphological versatility.

### 2.7. Microwave-Assisted Hydrothermal Synthesis

The defining feature of microwave-assisted hydrothermal synthesis is the rapid and uniform volumetric heating of the reaction medium, which reduces both processing time and energy consumption [124,125]. Its scientific and technological value therefore lies less in introducing a new phase-formation mechanism than in intensifying the conventional hydrothermal process.

Kostyukhin et al. [124] shortened the synthesis of LaFeO_3_ at 240 °C and 60 bar from 48 h to 3 h, accelerating the reaction by a factor of at least 16. However, the reported particle sizes of 70–100 nm [92] and 5–15 μm for cube-like particles [124] demonstrate that rapid synthesis does not necessarily yield nanoscale products. The primary advantage of this route is therefore its kinetic and energy efficiency, whereas additional parameter optimization is required to control morphology.

### 2.8. Alternative Methods for Preparing Orthoferrites

Beyond conventional powder synthesis, pulsed laser deposition (PLD) [126,127,128], spin coating (SCT) [129], electrospinning (EST) [130,131,132,133,134,135,136,137], and molecular beam epitaxy (MBE) [138,139] are employed to fabricate RFeO_3_ materials with defined geometries. The value of these techniques lies not simply in phase synthesis, but in their ability to create application-oriented architectures such as thin films and one-dimensional structures.

#### 2.8.1. Pulsed Laser Deposition (PLD)

PLD enables the stoichiometric composition of multicomponent perovskites to be transferred to a substrate with relatively high fidelity while allowing control over thin-film growth. It is therefore effective for producing high-quality functional films; however, its reliance on sophisticated vacuum and laser equipment, together with limited scalability, constrains industrial implementation [102].

In PLD, laser ablation of the target material, propagation of plasma species, deposition onto the substrate, and film growth through nucleation are interdependent stages [102,140]. Although the ability to preserve stoichiometry is a major advantage, film quality depends on laser energy, substrate temperature, and background pressure [141], requiring precise control of the processing window.

Cyza et al. [113] demonstrated the structural effects of Sr incorporation in La_1−x_Sr_x_FeO_3_ films, whereas Berini et al. [142] showed that the crystallinity of SmFeO_3_ films depended on laser fluence and substrate temperature. These findings indicate that chemical composition and growth parameters cannot be treated independently in PLD.

#### 2.8.2. Electrospinning (EST)

The principal advantage of electrospinning for RFeO_3_ systems is its capacity to produce one-dimensional nanofibers with high surface-to-volume ratios [133,137,143,144]. For sensing and surface-reaction-dependent applications, it therefore provides an alternative structural strategy to particulate powders.

The high aspect ratio, uniform diameter, and large specific surface area of electrospun fibers are important for sensing applications. Nanocomposite fibers with diameters as small as 10 nm can be obtained [145], although such dimensions are highly sensitive to solution composition and processing parameters.

Fiber morphology is governed by the properties of the polymer solution, applied voltage, flow rate, needle-to-collector distance, and ambient conditions [146]. Although this multivariable dependence increases the versatility of electrospinning, it also imposes stringent requirements on process reproducibility.

Jeong et al. [145] showed that, in Mn-doped LaFeO_3_ nanofibers, the diameter of the constituent particles decreased from 50 ± 5 nm in the pristine sample to 35 ± 5 nm at 15 mol.% Mn. This result demonstrates that not only the one-dimensional architecture but also doping can modify the size of the primary particles constituting the fibers.

### 2.9. Comparative Analysis of Synthesis Methods

Comparison of the available synthesis routes demonstrates that a simple list of “advantages and disadvantages” is insufficient for evaluating methods for RFeO_3_ preparation. The solid-state reaction is attractive in terms of simplicity and scalability, but processing at 900–1500 °C [49,50,54,57] and the risk of agglomeration and secondary-phase formation [98,99] limit precise control over nanoscale dimensions and morphology.

Sol–gel, coprecipitation, and hydrothermal routes provide enhanced chemical homogeneity and morphological control [80,86,87,89,102,103,104,117,118,119,120,121,122,123], but these benefits are accompanied by pronounced sensitivity to processing parameters. Thus, the potential to achieve low-temperature synthesis and fine particles yields reproducible results only when pH, precursor composition, gelation or precipitation, and calcination conditions are rigorously controlled.

Microwave-assisted hydrothermal synthesis is distinguished by its energy and time efficiency, reducing the LaFeO_3_ reaction time from 48 h to 3 h [124]. PLD, MBE, and electrospinning [126,127,128,129,130,131,132,133,134,135,136,137,138,139,143,144,145,146], by contrast, do not compete directly with powder-synthesis methods but are intended to produce specialized material forms, including thin films and one-dimensional architectures.

Consequently, no universally “best” synthesis method exists. The optical floating-zone method is preferred for single crystals; wet-chemical routes are advantageous for nanoscale materials with high specific surface areas; microwave-assisted hydrothermal synthesis is suitable when short reaction times are required; and specialized techniques are favored for device-integrable films or fibers. A comparative overview of the synthesis methods is presented in Table 1. Therefore, the principal criterion for evaluating a method is its ability to reproducibly achieve the structural parameters required for the intended function.

## 3. Structural and Property Characterization of Rare-Earth Orthoferrites

No single analytical technique is sufficient for the comprehensive characterization of RFeO_3_ orthoferrites. X-ray diffraction (XRD) and Rietveld refinement determine phase composition and crystallographic parameters; scanning and field-emission scanning electron microscopy (SEM/FESEM) and transmission and high-resolution transmission electron microscopy (TEM/HRTEM) reveal micro- and nanoscale morphology; energy-dispersive X-ray spectroscopy (EDS/EDX) determines elemental composition; Fourier-transform infrared spectroscopy (FTIR) probes chemical bonding; UV–visible spectroscopy identifies optical transitions; and vibrating-sample magnetometry (VSM) evaluates the magnetic response. The scientific value of these techniques lies not in their individual outputs, but in their complementary ability to establish composition–structure–morphology–property relationships.

Accordingly, the following analysis focuses less on the technical capabilities of the characterization methods themselves and more on how they elucidate the relationship between structural modification and functional response in RFeO_3_ systems.

### 3.1. Crystal Phase and Structure

**Figure 6:**
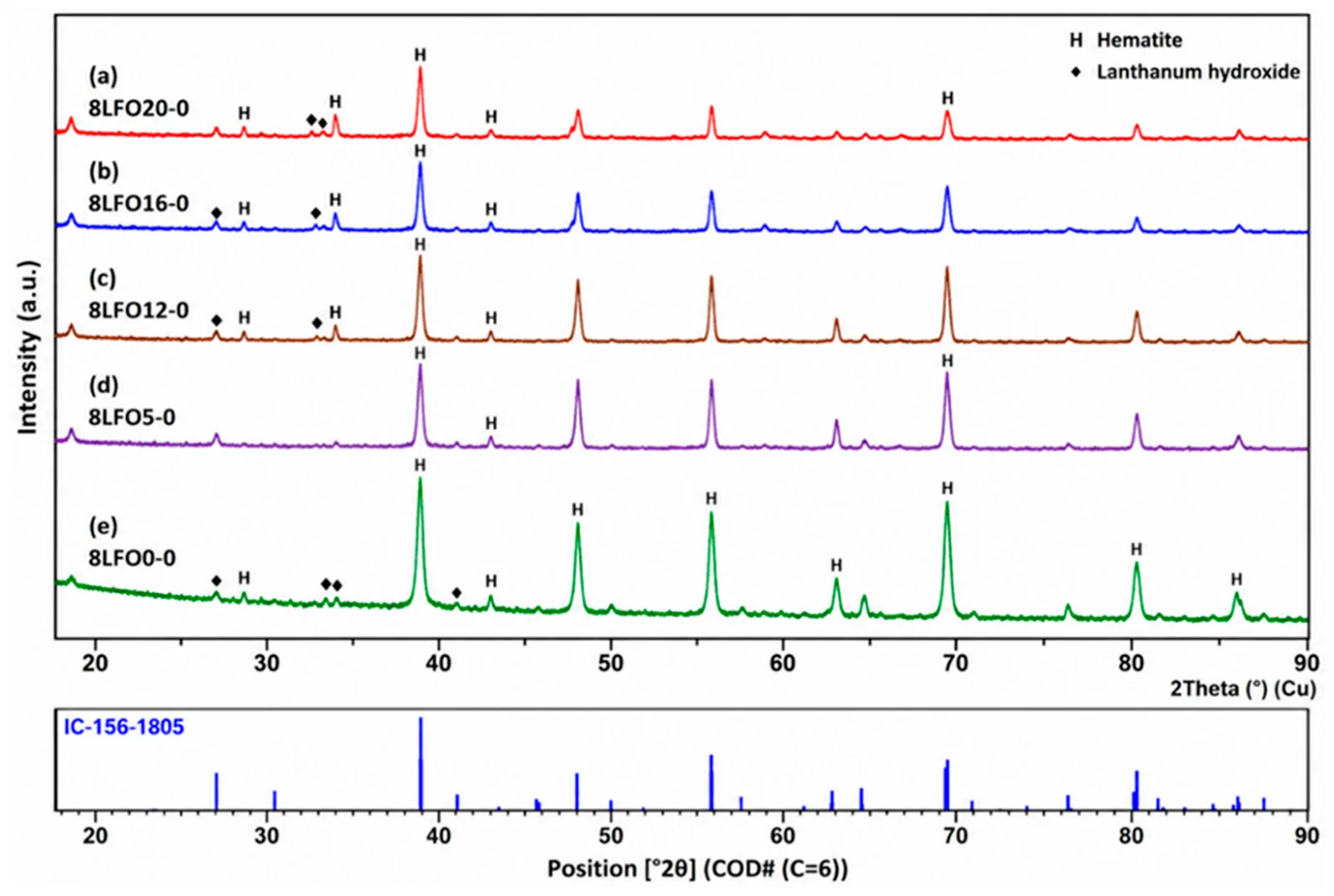
X-ray diffraction (XRD) patterns of the samples: (**a**) 0LFO12-0, prepared without mechanical activation; and mechanically activated samples (**b**) 2LFO12-0, (**c**) 8LFO12-0, (**d**) 8LFO16-0, and (**e**) 8LFO20-0. No additional mechanical milling was applied to these samples after the corresponding treatments. For comparison, the positions and relative intensities of the diffraction peaks of the LaFeO_3_ perovskite phase (CIF 96-156-1806) obtained from the PDF database are also shown.

XRD data for LaFeO_3_ demonstrate the influence of mechanical activation on phase-formation kinetics. As the milling time increased from 0 to 2 and 8 h, the intensity of the LaFeO_3_ reflections increased in Figure 6a–c. This finding indicates that mechanically induced dislocations, vacancies, and microstrain enhance the reactivity of the La_2_O_3_ and Fe_2_O_3_ precursors [148,149,150].

Extending the calcination time to 16 and 20 h increased the LaFeO_3_ fraction and produced a nearly pure perovskite structure, although minor hematite impurity phases remained (Figure 6d,e). These results demonstrate that mechanical activation accelerates phase formation, but subsequent thermal treatment is still required to achieve high phase purity.

**Figure 7:**
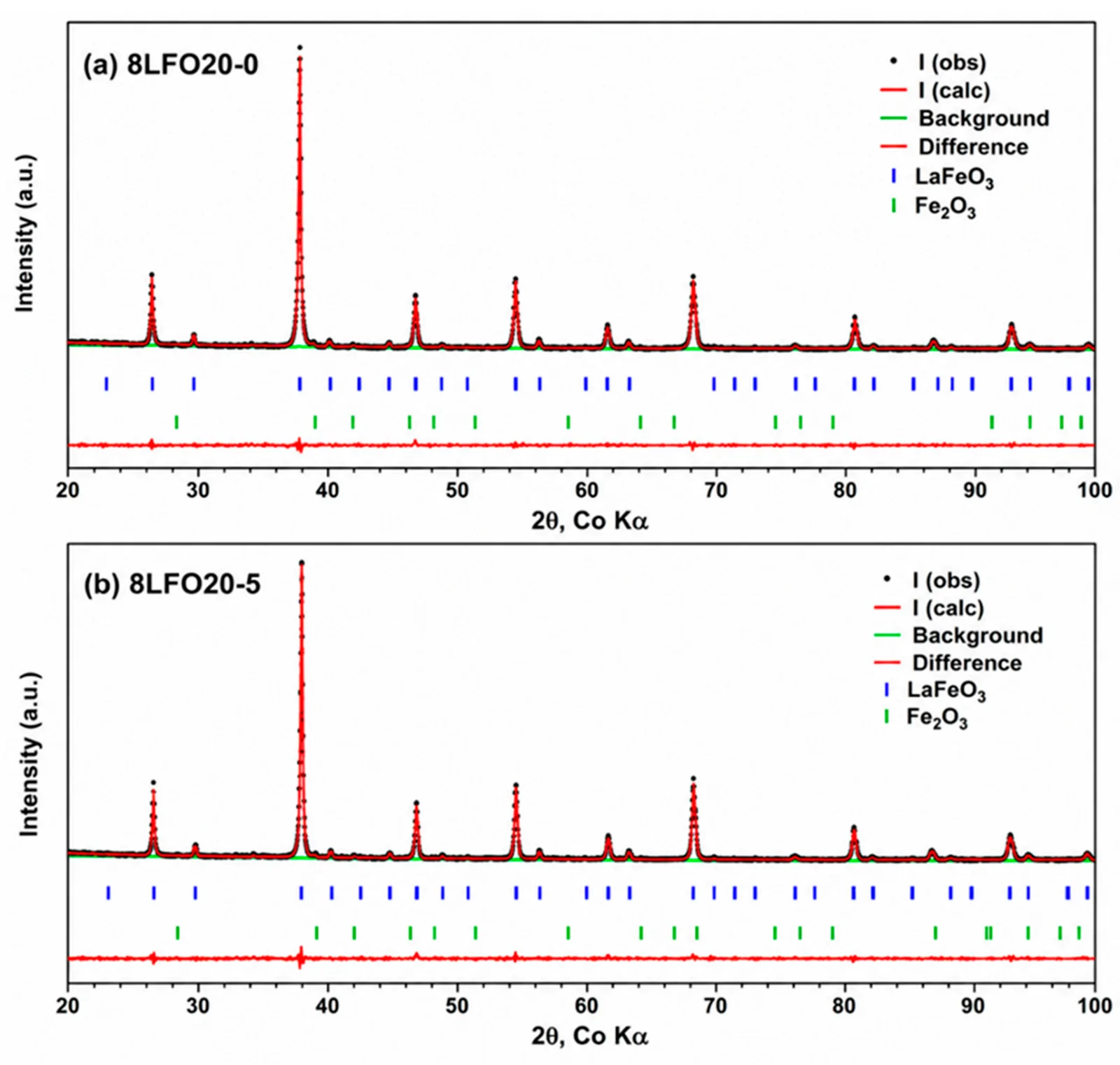
Rietveld profile refinements of the X-ray diffraction (XRD) patterns of the (**a**) 8LFO20-0 and (**b**) 8LFO20-5 samples.

The Rietveld refinement results presented in Figure 7 confirmed the orthorhombic structure of LaFeO_3_ in the Pnma space group and a phase purity of 97–98%. Although the crystallite size remained approximately 91–101 nm, the microstrain increased from 0.03% to 0.11% with increasing duration of mechanical treatment [151,152]. The structural parameter values are provided in Table 2. Minor changes in the lattice parameters were accompanied by a pronounced increase in microstrain.

### 3.2. FTIR Analysis

**Figure 8:**
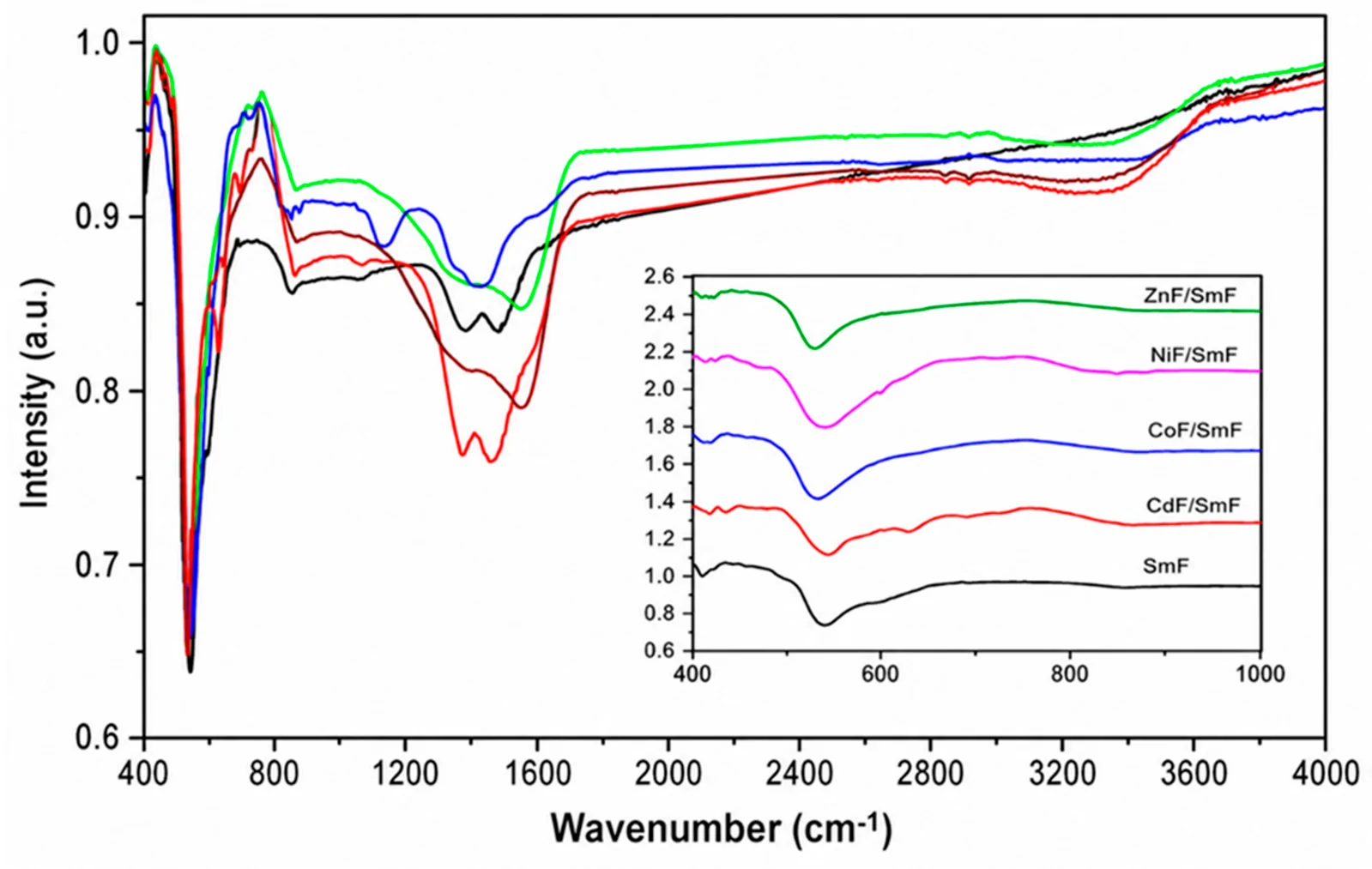
FTIR spectra of SmFeO_3_ and MF/SmFeO_3_ nanocomposites.

FTIR data enable the evaluation of metal–oxygen bonding and interfacial interactions in SmFeO_3_ and MF/SmFeO_3_ systems. The bands in the 1000–400 cm^−1^ region correspond to lattice metal–oxygen vibrations (Figure 8), and their positions and widths are sensitive to the cationic environment and structural distortion [153,154]. The spectra should therefore be used not merely for band assignment, but also as an independent means of verifying the phase and structural changes identified by XRD.

In SmFeO_3_–MFeO_4_ nanocomposites, shifts and broadening of the Fe–O bands have been attributed to interfacial strain, lattice distortion, and cation redistribution [153,154,155,156,157,158]. The shift in bands from 1384–1491 cm^−1^ to the 1428–1565 cm^−1^ region in the composites is likewise consistent with interactions between the constituent phases. Such spectral shifts should not, however, be treated as stand-alone evidence; when considered together with XRD and microstructural findings, they provide a more reliable indication of structural interactions.

### 3.3. XPS Analysis

**Figure 9:**
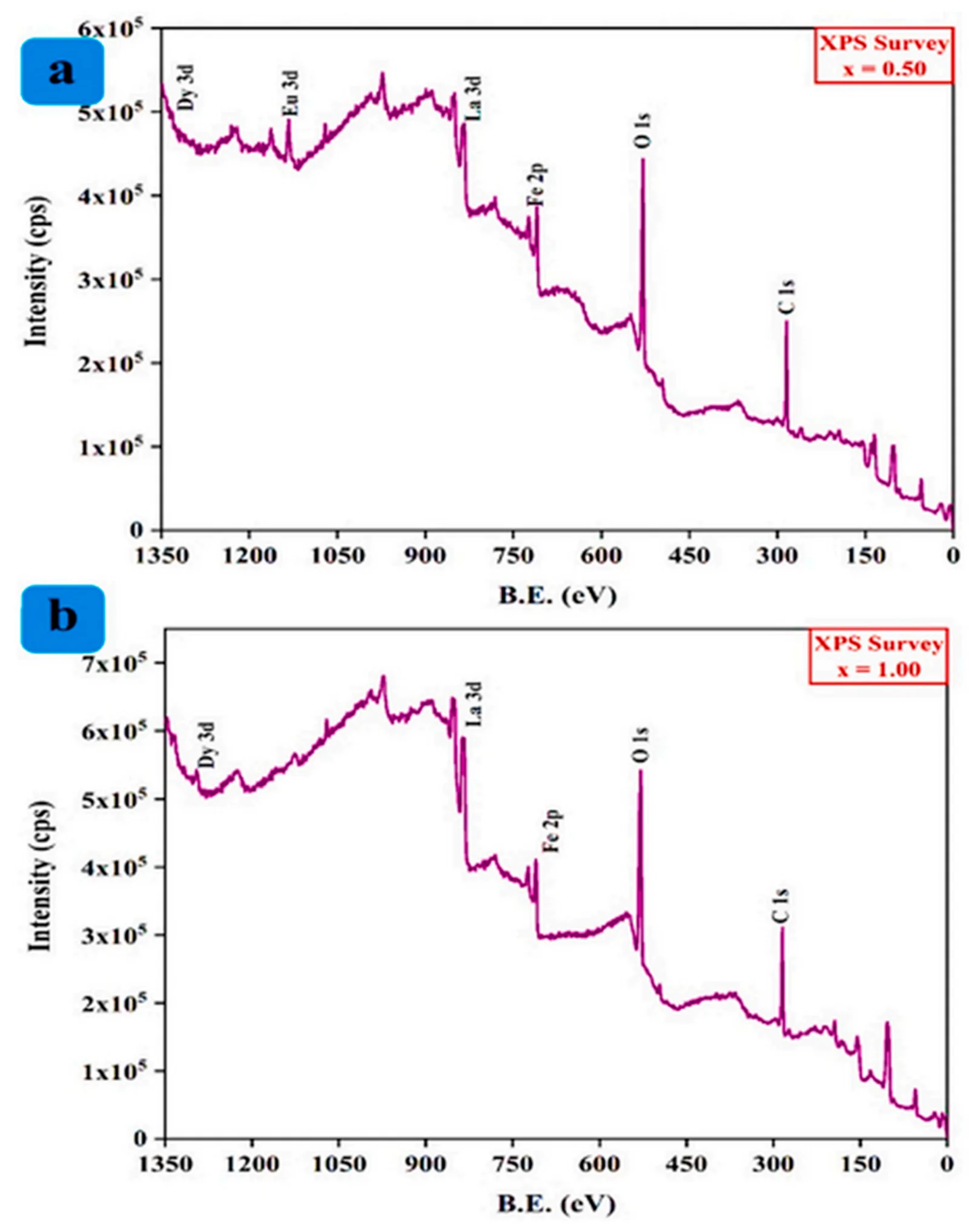
X-ray photoelectron survey spectra of the (1 − x)Eu_3_Fe_5_O_12_–(x)La_0.5_Dy_0.5_FeO_3_ composite: (**a**) x = 0.50 and (**b**) x = 1.00.

The elemental composition and surface chemical states of the Eu_3_Fe_5_O_12_–La_0.5_Dy_0.5_FeO_3_ system were investigated by X-ray photoelectron spectroscopy. Figure 9 presents survey spectra recorded over the binding-energy range of 1400–0 eV. Signals from La, Dy, Eu, Fe, and O were detected in the biphasic LDE3 (x = 0.50) sample, whereas La, Dy, Fe, and O were identified in the single-phase LDE5 (x = 1.00) sample. The absence of an Eu signal in the LDE5 spectrum is consistent with its La_0.5_Dy_0.5_FeO_3_ composition. The C 1s peak near 284.8 eV was used to calibrate the binding-energy scale [159]. The absence of detectable impurity peaks within the instrumental sensitivity limit indicated the high chemical purity of the samples.

The chemical states of the elements were further resolved from the high-resolution La 3d, Dy 3d, Eu 3d, Fe 2p, and O 1s spectra presented in Figure 6. The spectra were fitted with Voigt functions using Krefting v1.70 software, with a U2-Tougaard background applied in most cases. A linear background was used for the La 3d spectrum of the single-phase sample [160].

**Figure 10:**
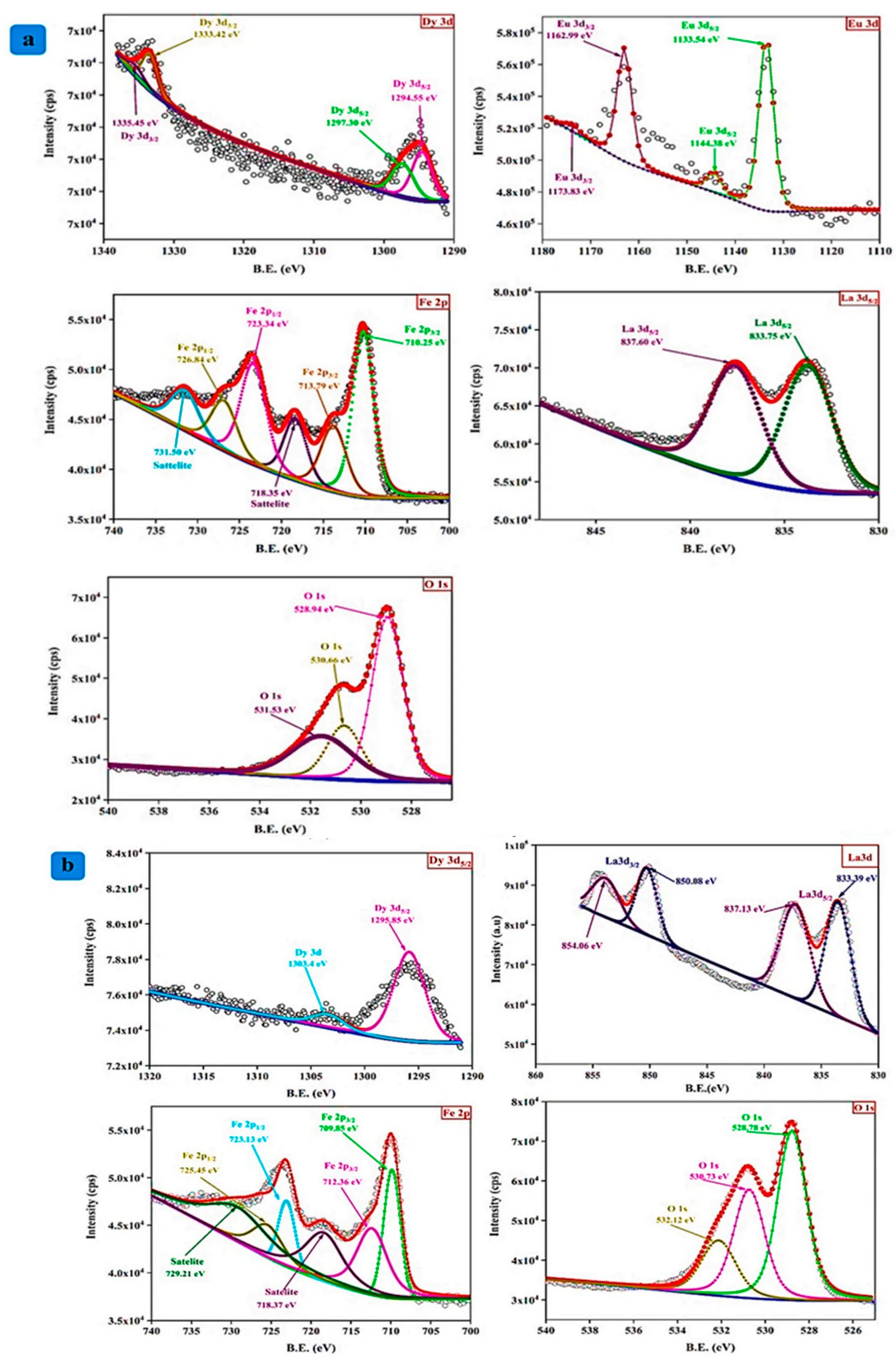
Deconvoluted XPS peaks of the (1 − x)Eu_3_Fe_5_O_12_–(x)La_0.5_Dy_0.5_FeO_3_ composite: (**a**) x = 0.50 and (**b**) x = 1.00.

The Dy 3d_5_/_2_ peak located at approximately 1295.8 eV in Figure 10a,b confirmed the Dy^3+^ oxidation state [161,162]. The Eu 3d_5_/_2_ and Eu 3d_3_/_2_ components were observed at 1133–1134 and 1163–1164 eV, respectively. The absence of shoulders characteristic of Eu^2+^ indicated that europium was present predominantly as Eu^3+^ [163]. Although the Eu 4d signal in the 135–146 eV region provided additional support for this conclusion, the survey-spectrum data were insufficient for quantitative analysis and rigorous component deconvolution [164].

The principal and satellite La 3d_5_/_2_ peaks were recorded at 833.75 and 837.60 eV, respectively, while the La 3d_3_/_2_ peaks occurred near 850.08 and 854.06 eV. These features are characteristic of La^3+^ and originate from charge-transfer phenomena [165]. In Figure 6a,b, the Fe 2p_3_/_2_ peaks were observed at 710.25 and 709.85 eV, whereas the shake-up satellite occurred at 718.35–718.37 eV, confirming the presence of Fe^3+^ ions [166]. The Fe 2p_1_/_2_ component was identified at approximately 724.3 eV [167].

The O 1s peaks at 528.94 and 528.78 eV were assigned to lattice oxygen. The component at 530.66–530.73 eV was attributed to oxygen associated with surface defects, oxygen vacancies, or hydroxyl species [168]. The component at 531.53–532.12 eV corresponded to adsorbed oxygen species and surface oxygen-containing groups [169]. Minor differences in the relative intensities of these components indicated subtle composition-dependent variations in surface oxygen chemistry.

Overall, the predominance of La^3+^, Dy^3+^, Eu^3+^, and Fe^3+^ states was consistent with the XRD, Raman, and VSM results. The authors associated the predominance of Fe^3+^ with the observed weak-ferromagnetic and spin-canted antiferromagnetic behavior. The absence of reduced iron species and secondary oxides was regarded as evidence that the magnetic response arose from the intrinsic properties of the material.

### 3.4. Raman Spectroscopic Analysis

Raman spectroscopy was employed over the wavenumber range of 100–700 cm^−1^ to investigate local structural transformations and phase coexistence in the EIG–LDF system (x = 0.00–1.00). The analysis primarily focused on phase identification and structural confirmation. While X-ray diffraction provides information on long-range order within the crystal structure, Raman scattering is sensitive to lattice distortions, octahedral tilting [170,171], short-range order, and interfacial strain [172]. These characteristics make Raman spectroscopy particularly suitable for probing the local structure of garnet–perovskite composites.

The Raman spectrum of the LDE1 (x = 0.00) sample shown in Figure 10 exhibits well-defined vibrational modes characteristic of the cubic garnet structure (Ia-3d). The low-frequency bands observed at approximately 140–160 cm^−1^ are attributed to translational lattice vibrations of Eu^3+^ ions relative to the rigid Fe–O framework [173]. The modes in the range of approximately 220–280 cm^−1^ are assigned to translational vibrations of the sublattices associated with FeO_6_ octahedra and FeO_4_ tetrahedra [174]. The intermediate-frequency modes between 300 and 500 cm^−1^ exhibit a mixed character, involving internal vibrations of rare-earth ions, iron ions occupying tetrahedral sites, and oxygen atoms, as well as coupled internal and rotational vibrations of the tetrahedral units [175,176]. The high-frequency band located at approximately 560–580 cm^−1^ corresponds to the stretching vibrations of Fe–O bonds within the garnet structure [177]. These spectral features confirm the high phase purity and pronounced degree of local structural ordering, consistent with the single-phase nature and fine-grained microstructure of the sample.

As the LDF content increases from x = 0.25 to x = 0.75, the vibrational modes characteristic of the perovskite phase progressively become more pronounced, whereas the spectral features associated with the garnet phase gradually weaken. The bands emerging at approximately 150–180 and 250–300 cm^−1^ are assigned to the lattice vibrations of La/Dy ions at the A sites of the orthorhombic perovskite phase (Pbnm) and to the rotational modes of FeO_6_ octahedra, respectively [178]. The intense modes observed in the approximately 400–450 and 600–650 cm^−1^ regions correspond to O–Fe–O bending and Fe–O stretching vibrations within tilted FeO_6_ octahedra [179]. The simultaneous presence of garnet- and perovskite-related modes within the same spectrum confirms the formation of a two-phase composite. This finding is consistent with the phase fractions determined by Rietveld refinement.

**Figure 11:**
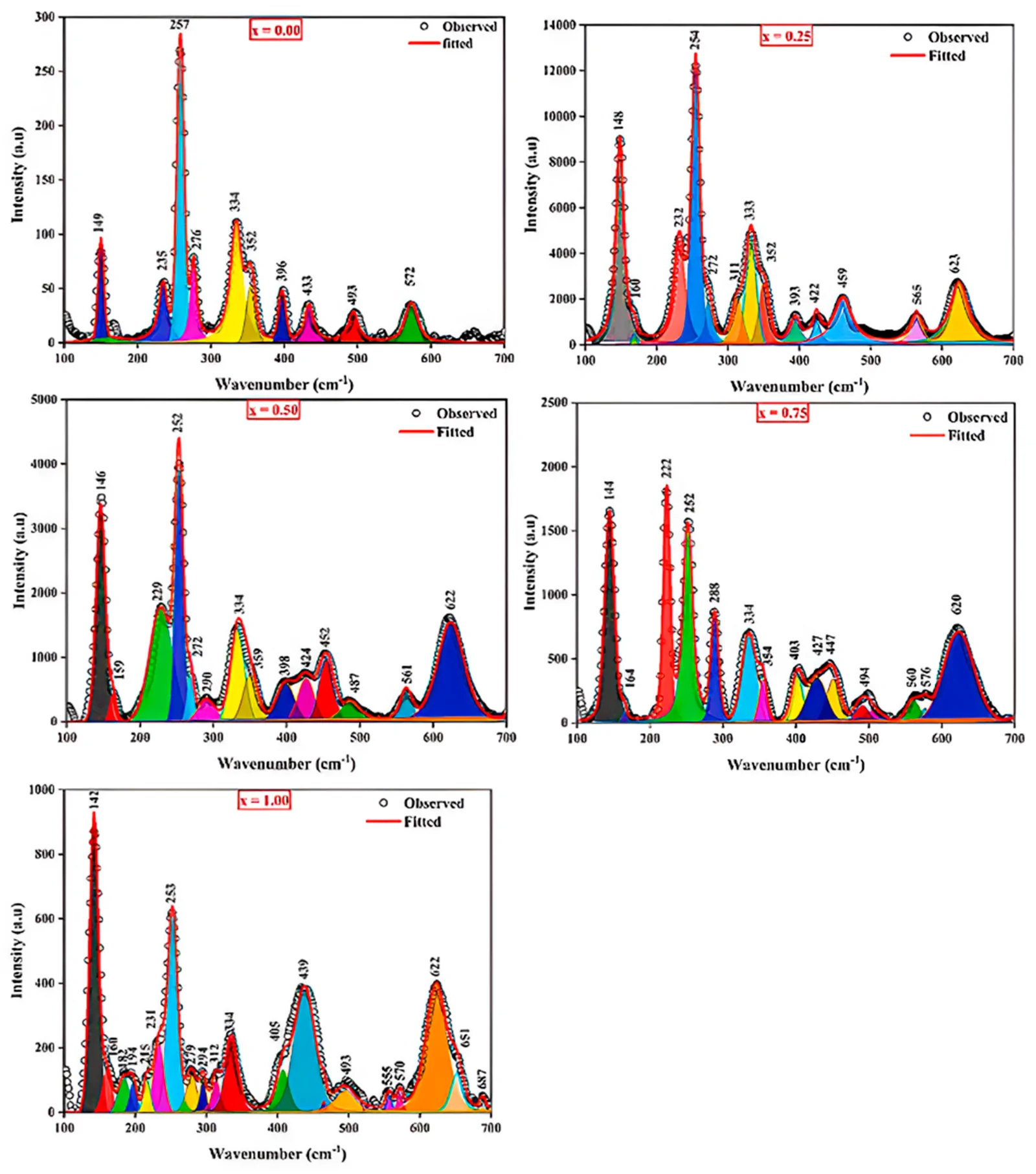
Raman spectra of the EIG–LDF composite with different compositions.

In particular, the pronounced broadening of the Raman peaks observed for the x = 0.50 composition is attributed to interfacial strain at the garnet–perovskite boundaries, variations in crystallite size, and phonon-confinement effects [180]. The slight shifts in the Raman-band positions may be associated with local lattice distortions induced by the coexistence of the two phases. For the LDE5 (x = 1.00) sample, only the vibrational modes characteristic of the perovskite phase remain, whereas the garnet-related features completely disappear. This result confirms the formation of a single-phase La_0.5_Dy_0.5_FeO_3_ structure. As shown in Figure 11, the experimental Raman spectra (open black circles) are deconvoluted into individual vibrational components, whose sum forms the overall fitted curve represented by the solid red line. Red, green, blue, cyan, yellow, orange, and purple shading distinguishes the overlapping spectral components.

### 3.5. Morphological Analysis

The complementary use of SEM/FESEM, TEM/HRTEM, and selected-area electron diffraction (SAED) is essential in morphological analysis because functional properties are affected not only by particle size and shape, but also by porosity, agglomeration, crystallographic state, and defects [70,87,95,108,122,131,134,181]. Microscopy images should therefore be treated not merely as illustrations, but as a means of quantitatively evaluating synthesis–microstructure–property relationships.

Statistical analysis of size distributions using Image11 and Nano Measurer [70,79,98], together with verification of elemental composition and spatial homogeneity by EDS [84,115,122], improves the reliability of comparisons among samples prepared by different synthesis routes. This approach is particularly important for distinguishing materials with similar mean particle sizes but different degrees of agglomeration and elemental homogeneity.

**Figure 12:**
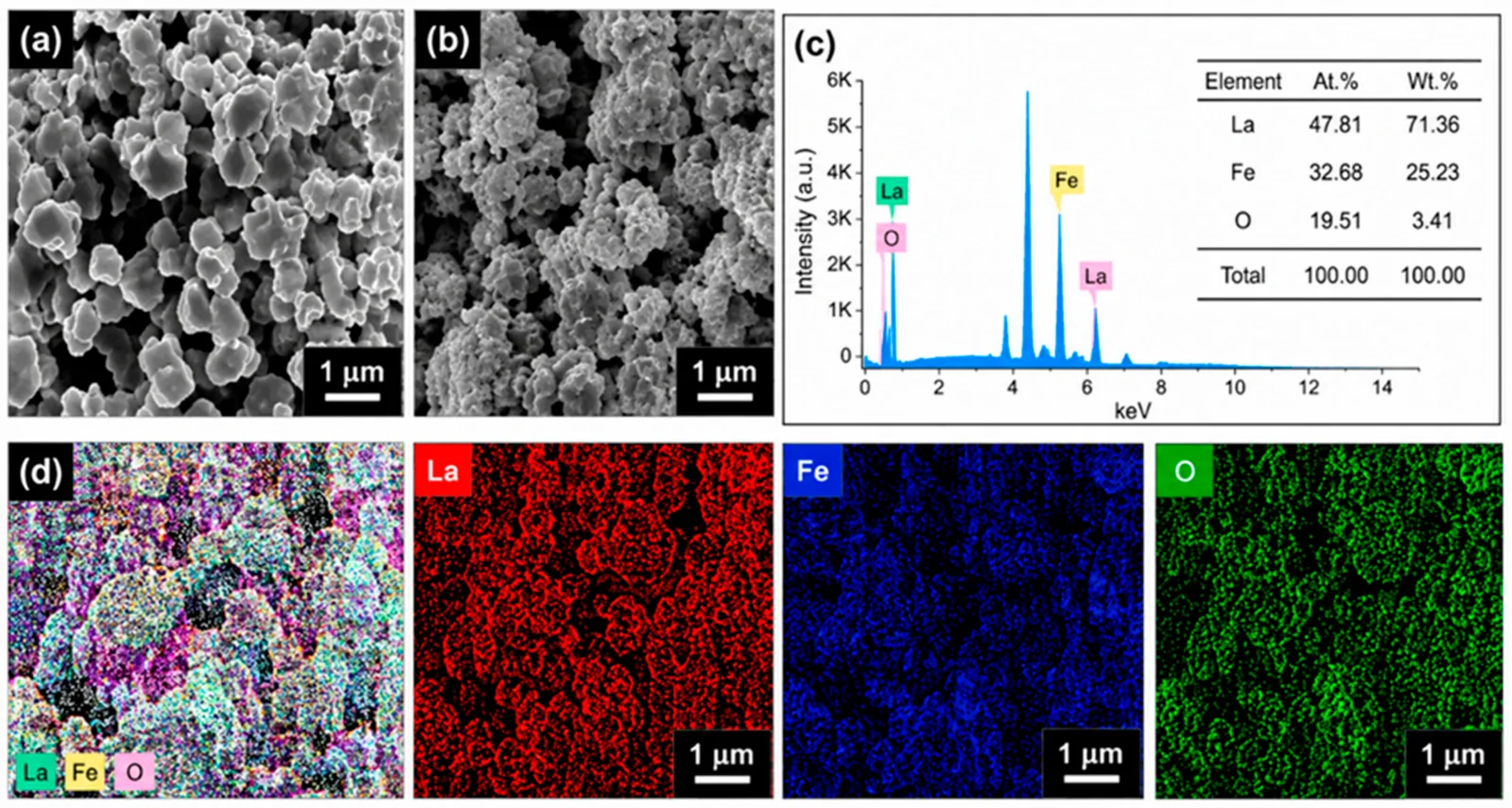
(**a**) Micrograph of the surface morphology of a LaFeO_3_ (LFO) pellet; (**b**) micrograph of the surface morphology of LFO particles; (**c**) EDS spectrum of the LFO sample showing atomic fractions (at.%); and (**d**) elemental maps showing the presence and distribution of La, Fe, and O in the resulting LFO.

In the solid-state-derived LaFeO_3_ sample investigated by Lee et al. [182], a polycrystalline microstructure with clearly discernible grains and grain boundaries, together with surface pores, was observed (Figure 12a,b). Such porosity may facilitate interactions between gas molecules and the material surface. The EDS spectrum and elemental mapping confirmed the presence and homogeneous distribution of La, Fe, and O (Figure 12c,d). Concurrent assessment of morphology and elemental homogeneity therefore avoids attributing sensing potential to porosity alone.

Published data indicate that sol–gel-derived RFeO_3_ (R = La, Pr, Nd, Sm, and Gd) particles are generally agglomerated and approximately spherical. A decrease in the R-cation radius may be accompanied by smaller particle sizes and improved morphological uniformity [183]. Although this trend indicates that the identity of the rare-earth cation influences the microstructure, it should not be regarded as universal because broad size distributions of 45–252 nm have been reported for Dy-, Ho-, and Er-based systems [70,107].

The observation of spherical grains with relatively limited agglomeration in NdFeO_3_ [70,107] suggests that the influence of the R cation is coupled to the synthesis conditions. Comparisons of the morphology of different RFeO_3_ compositions must therefore consider not only the cation radius but also equivalent synthesis and thermal-treatment conditions.

In some studies, hydrothermal synthesis produced more uniform and larger particles than the sol–gel method, accompanied by improved magnetic characteristics [89]. Microwave-assisted hydrothermal synthesis of LnFeO_3_ (Ln = Nd, Dy, and Er) yielded weakly agglomerated particles of 70–100 nm [92]. These findings demonstrate the inadequacy of the simple assumption that smaller particles invariably produce better properties and highlight the need to evaluate crystallinity and morphology together.

In solid-state synthesis, increasing the sintering temperature promotes grain growth [98], whereas coprecipitation and combustion methods can produce agglomerated, flake-like, or porous morphologies [104]. These differences indicate that the final microstructure is governed not only by chemical composition but also by the reaction pathway of the precursors and subsequent growth kinetics.

Electrospinning enables the fabrication of one-dimensional porous structures with rough surfaces that are fundamentally different from particulate morphologies [134]. Such architectures may increase the accessibility of surface-active sites; therefore, their advantage lies more in their surface-to-volume ratio and transport-path geometry than in their mean particle size.

The effect of doping on morphology is not unidirectional. Increasing the Cr content enlarged SmFeO_3_ grains to 0.4–2 μm [184], whereas increasing the Ce content reduced LaFeO_3_ particle size and improved morphological uniformity [85]. These contrasting trends demonstrate that the effect of a dopant cannot be interpreted independently of its identity, concentration, and the synthesis method.

TEM/SAED confirmation of weakly agglomerated particles of 39–77 nm in the LaFe_1−x_Ti_x_O_3_ system [84] supports the influence of B-site substitution on microstructure. To correlate morphological modification with functional properties, however, changes in phase purity and defect state must be monitored alongside doping.

For practical gas-sensing applications, high porosity and loosely bound agglomerates can enhance gas-molecule diffusion and increase the available surface-interaction area [116,185]. Accordingly, the morphology required for effective sensing should provide a balance between accessible porosity and stable conduction pathways rather than maximum density.

The preparation of spherical DyFeO_3_ particles of 20–70 nm [117], cube-like hydrothermal SmFeO_3_ particles of approximately 20 μm [186], and porous electrospun NdFeO_3_ nanorods [134] demonstrates that synthesis can tune RFeO_3_ morphology over a wide range, from nanoparticles to microcubes and one-dimensional structures. Thus, the primary criterion in morphological assessment should not be “fineness,” but rather compatibility of the geometry with the intended function.

Overall, the RFeO_3_ microstructure is governed by the interplay among synthesis method, chemical composition, dopant concentration, and thermal treatment [89,116,134,184,185]. Rather than characterizing morphological parameters in isolation, their causal relationships with dielectric, magnetic, and sensing responses should therefore be examined within the same set of samples.

### 3.6. Dielectric Properties of Rare-Earth Orthoferrites

**Figure 13:**
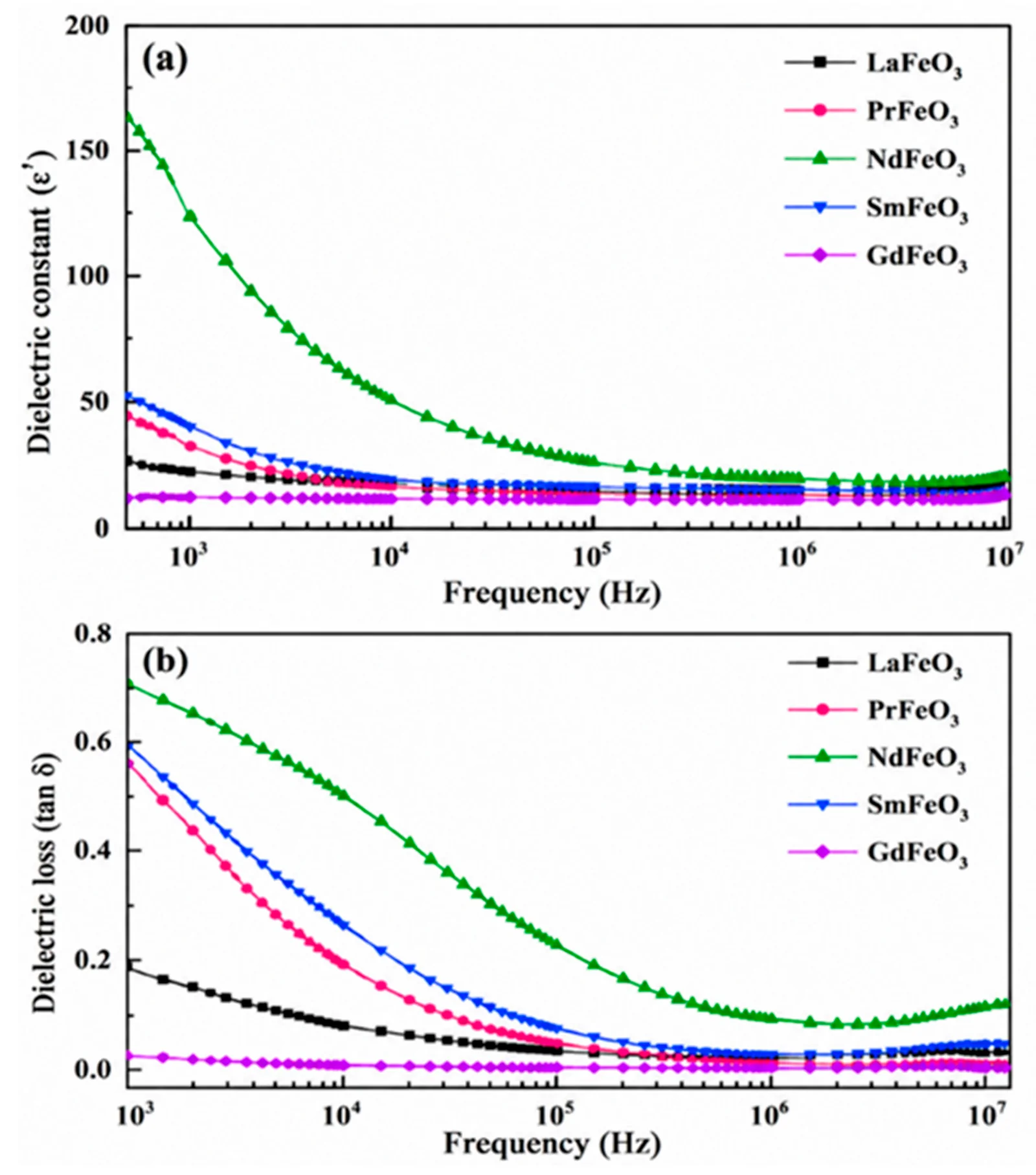
Frequency dependence of (**a**) dielectric permittivity (ε′) and (**b**) dielectric loss (tan δ) for RFeO_3_ (R = La, Pr, Nd, Sm, and Gd) orthoferrites.

For RFeO_3_ (R = La, Pr, Nd, Sm, Gd, Dy, Ho, Yb, and Lu) systems, both ε′ and tan δ exhibit a decreasing trend with increasing frequency over the 500 Hz–10 MHz range [150,187] (Figure 13a,b). The Maxwell–Wagner and dipolar-relaxation models explain this behavior in terms of the contributions of space-charge/dipolar polarization at low frequencies and faster polarization mechanisms at higher frequencies [94]. However, attributing ε′ solely to frequency is insufficient because FeO_6_ distortion and particle size are also relevant parameters [150,188].

Na-doped GdFeO_3_ exhibits an exceptionally pronounced increase in ε′ at low frequencies. At 50 Hz, ε′ is approximately 30 for the pristine sample but reaches values on the order of 10^6^ for the doped samples; at 10 kHz, the values are 934, 4748, and 8001 for x = 0.05, 0.1, and 0.2, respectively [112]. This substantial enhancement cannot be attributed solely to an “intrinsic” dielectric effect of doping, but also reflects increased contributions from mobile charge carriers and interfacial/space-charge polarization. For application-oriented assessment, the giant low-frequency ε′ values must therefore be interpreted together with dielectric-loss and impedance data.

The shift in the relaxation maximum in Figure 13b toward higher frequencies with increasing Na concentration, accompanied by a reduction in low-frequency tan δ [112], indicates that charge-transport dynamics are composition-sensitive. Thus, the effectiveness of doping should be assessed not only in terms of enhanced ε′ but also through changes in relaxation frequency and dielectric loss.

The AC electrical conductivity is expressed as:

σac = ωε0ε′tanδ (1)

**Figure 14:**
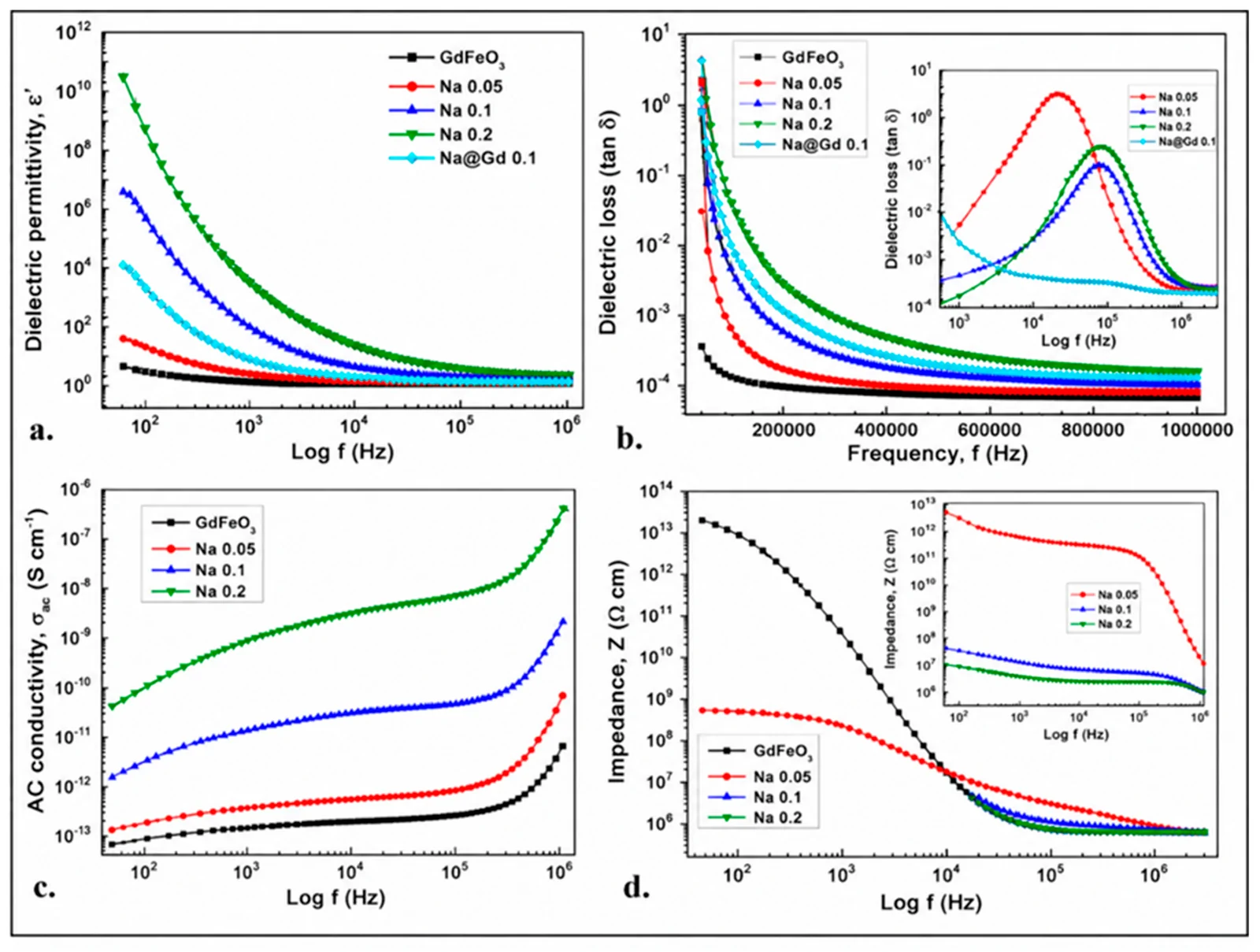
Frequency dependence of (**a**) dielectric permittivity (ε′), (**b**) dielectric loss (tan δ), (**c**) AC electrical conductivity (σₐ), and (**d**) impedance (Z) for GdFeO_3_ and Na-doped GdFeO_3_ samples at room temperature.

Figure 14 show an increase in AC conductivity and a decrease in impedance following Na incorporation [112]. These changes are consistent with enhanced hopping transport involving charge carriers and oxygen vacancies and underscore the importance of accounting for the conductivity contribution to high ε′ values.

The results summarized in Table 3 demonstrate that the dielectric response of RFeO_3_ is governed by the combined effects of frequency, structure, and composition. Na doping in GdFeO_3_ increases the real part of the dielectric permittivity (ε′) while reducing dielectric loss and impedance within a specific frequency range. Nevertheless, the contributions of conductivity and relaxation processes must be distinguished before high dielectric permittivity can be considered a practical advantage.

### 3.7. Optical Properties

**Figure 15:**
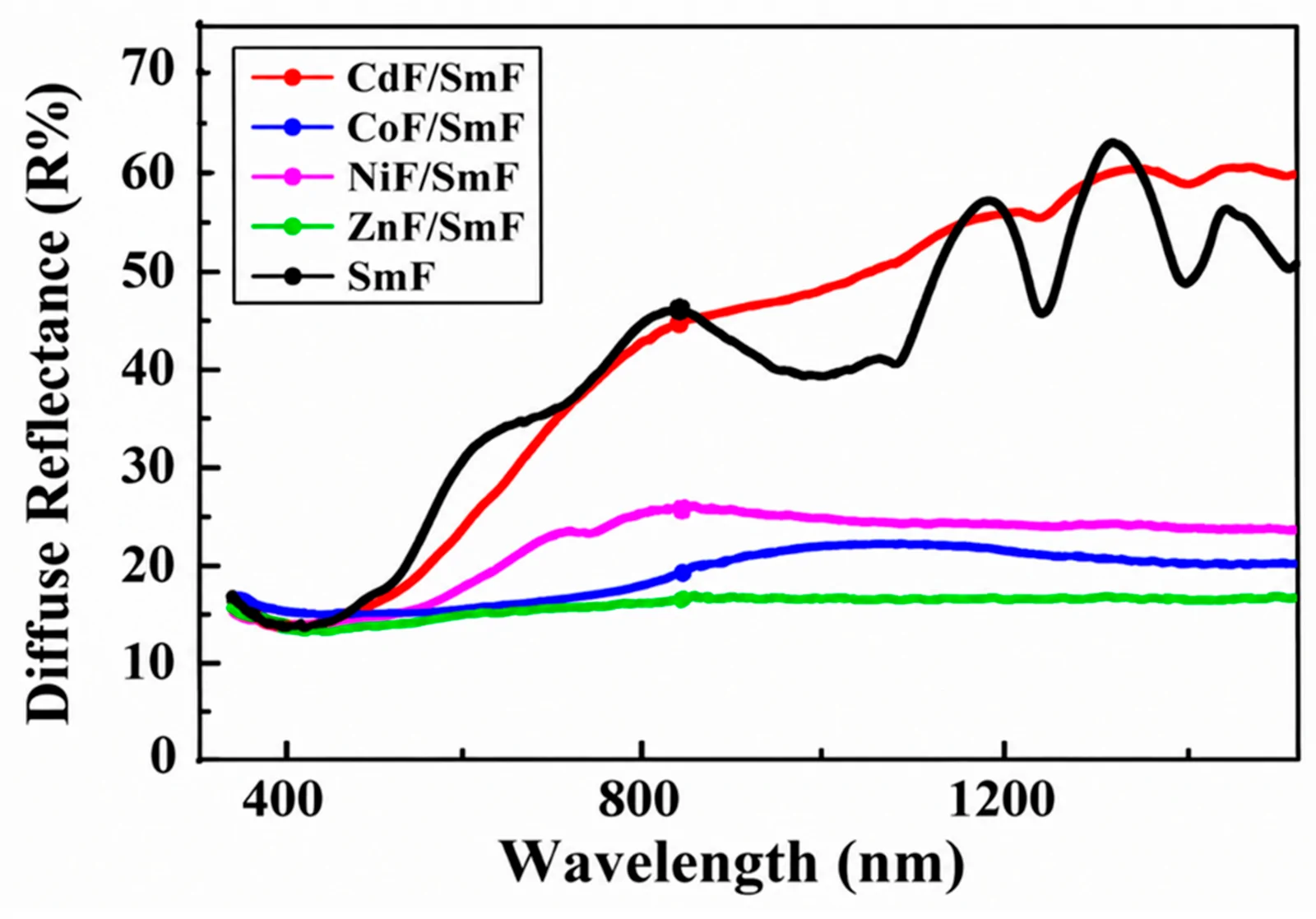
Optical reflectance spectra of SmFeO_3_ and MF/SmFeO_3_ nanocomposites.

The UV–visible spectra of MF/SmFeO_3_ nanocomposites were examined over the wavelength range of 300–1500 nm, and the band-gap energy was estimated using the Tauc method (Figure 15) [198]. The primary purpose of this analysis was not merely to report individual $E_g$ values, but to compare the extent to which the secondary ferrite phase modifies the electronic structure of SmFeO_3_.

(αhν)1/γ = B(hν − Eg) (2)

where h is Planck’s constant, ν is the photon frequency, Eg is the band-gap energy, and B is a constant. The coefficient γ depends on the type of electronic transition and is equal to 1/2 for a direct transition and 2 for an indirect transition [198]. The band-gap energy was determined by extrapolating the linear region of the Tauc plot of (αhν)^2^ versus hν.

The obtained Eg values were 2.28 eV for SmFeO_3_ and 1.18, 1.80, 1.72, and 2.00 eV for CoF/SmF, NiF/SmF, ZnF/SmF, and CdF/SmF, respectively [198,199,200]. The reduction in Eg in the composites extends their visible-light absorption range. However, the lowest Eg does not automatically correspond to the highest photocatalytic efficiency; charge separation, recombination, and interfacial transport must also be considered.

Variations in the reported Eg values have been attributed to particle size, morphology, the nature of the incorporated oxide, and possible quantum-confinement and interfacial effects [201,202,203,204,205,206]. Direct comparison of Eg values across different studies therefore requires consistent measurement conditions and Tauc-extrapolation procedures.

### 3.8. Magnetic Hysteresis Loops (VSM)

**Figure 16:**
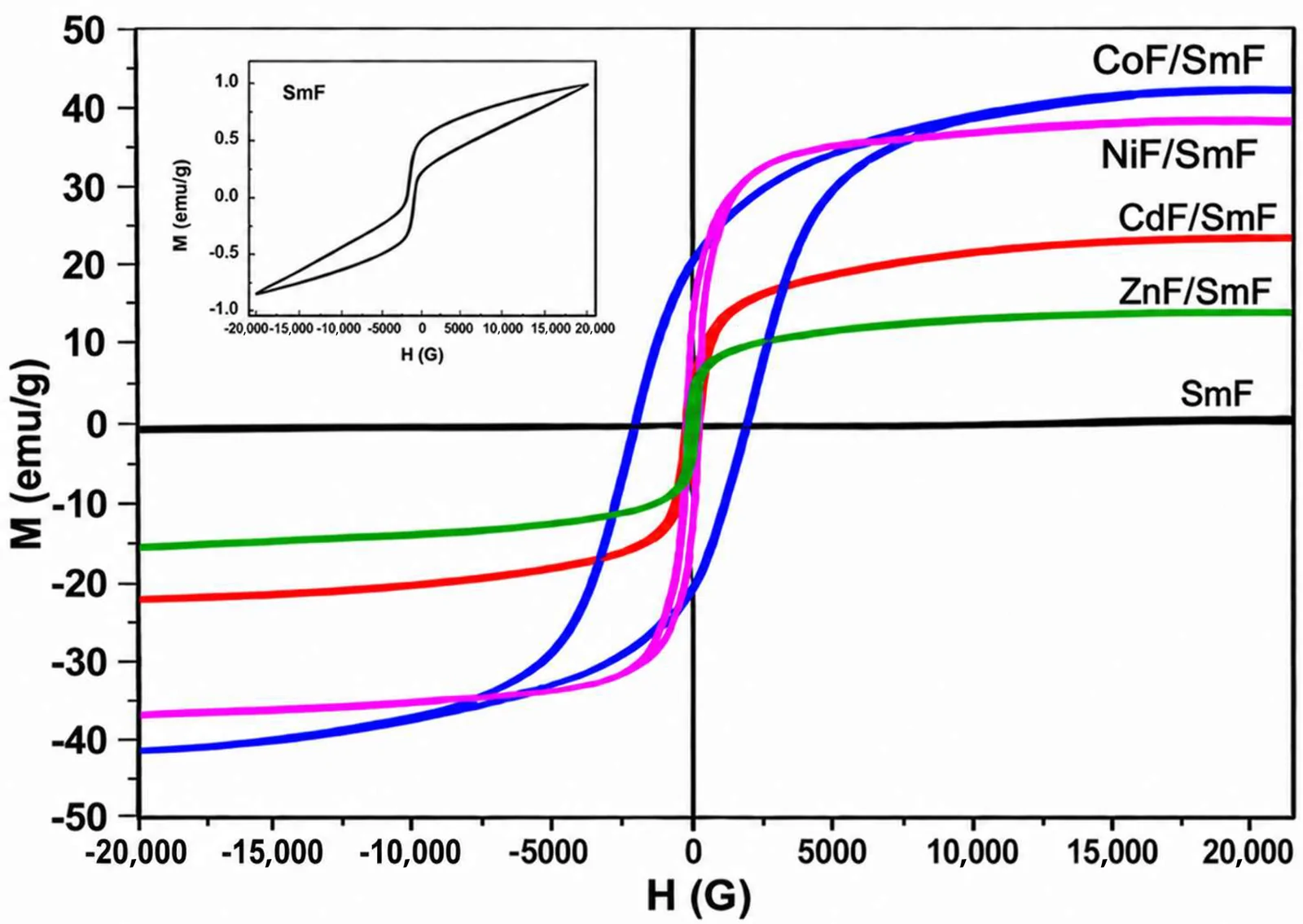
M–H magnetic hysteresis loops of SmFeO_3_ and MF/SmFeO_3_ nanocomposites.

As shown in Figure 16, the magnetic response of MF/SmFeO_3_ systems reflects the combined effects of cation distribution, crystallite size, morphology, and interfacial interactions. For SmFeO_3_, measurements performed under an applied magnetic field of 20 kOe yielded M_s_ = 0.796 emu g^−1^, M_r_ = 0.209 emu g^−1^, and H_c_ = 505.8 Oe. These parameters were associated with an antiferromagnetic state containing a weak ferromagnetic component arising from Fe^3+^ spin canting [16,207]. Composite formation may further influence magnetic exchange interactions by modifying FeO_6_ octahedral tilting and Fe–O–Fe bond angles.

An M_r_/M_s_ ratio below 0.5 is consistent with a predominantly multidomain state in the samples (Table 4). For CoF/SmFeO_3_ particles with a size of 33 nm, H_c_ = 1941 G and the high magnetocrystalline anisotropy indicate an approach toward a single-domain state. This distinction highlights the need to interpret magnetic properties in terms of domain state and particle size as well as chemical composition. The magnetic moment in Bohr magnetons (μB), magnetocrystalline anisotropy constant (K), and initial magnetic permeability (μ_i_) are also reported in Table 4 [208].

Variations in M_s_, M_r_, and H_c_ among the MF/SmFeO_3_ samples have been attributed to changes in magnetocrystalline anisotropy, domain structure, and superexchange interactions [209,210,211,212]. It is therefore more appropriate to interpret the magnetic enhancement following composite formation by considering concurrent changes in cation distribution, particle size, and Fe–O–Fe geometry rather than attributing it to a single interfacial effect.

### 3.9. Voltammetric and Impedance Evaluation of Electrocatalytic Activity

The electrocatalytic properties of the La_0.9_MnO_3_–LaFeO_3_ nanocomposite toward the hydrogen evolution reaction (HER) were investigated by linear sweep voltammetry (LSV) and electrochemical impedance spectroscopy (EIS). The polarization curves enabled the dependence of catalyst activity on cycle number to be evaluated, while impedance measurements provided insight into charge-transfer processes at the electrode–electrolyte interface.

**Figure 17:**
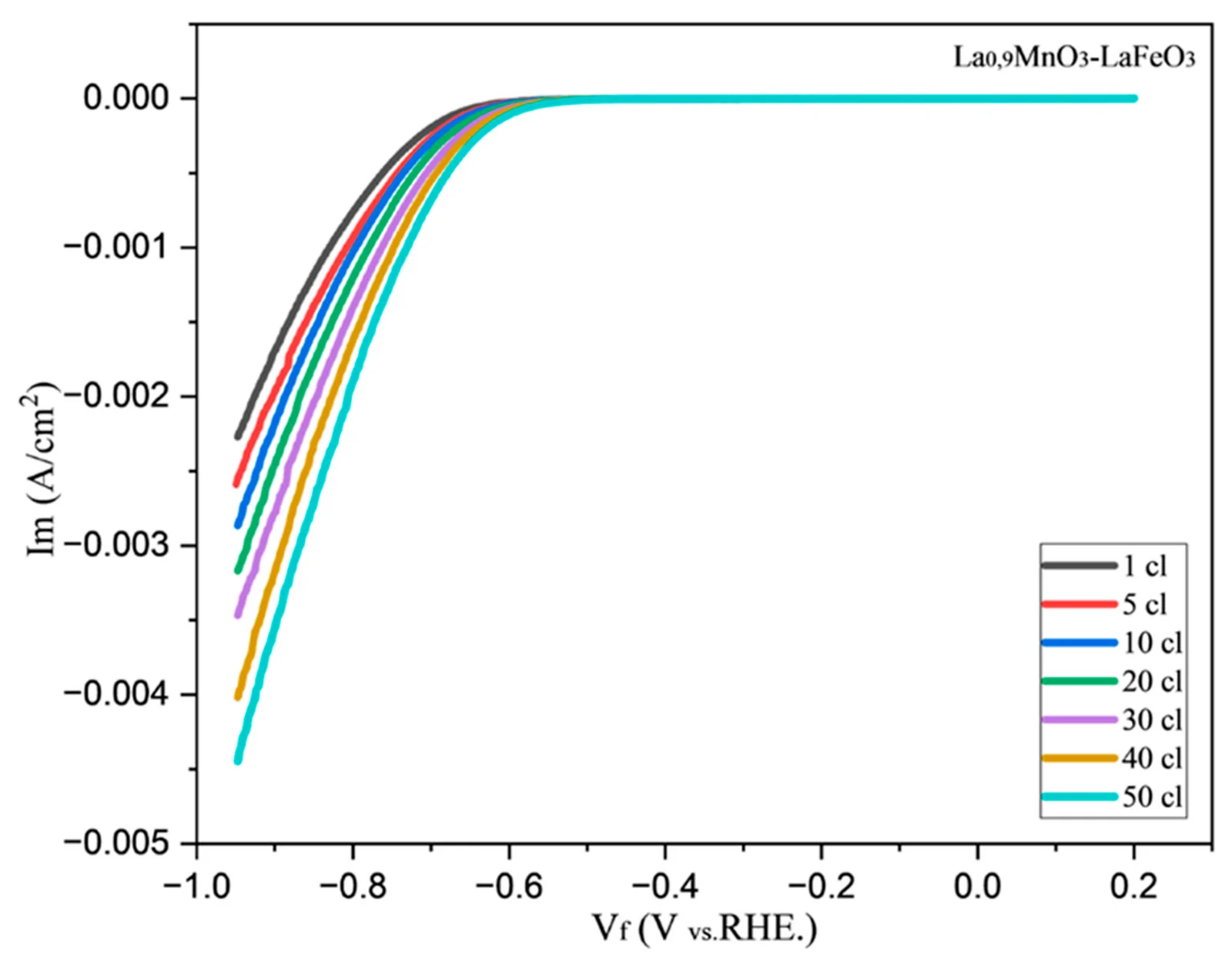
LSV curves of the La_0.9_MnO_3_–LaFeO_3_ nanocomposite.

The LSV curves presented in Figure 17 were recorded in a three-electrode system containing 0.5 M H_2_SO_4_ over a potential range of 0.2–0.95 V versus an Ag/AgCl reference electrode. The scan rate was 9.99 mV s^−1^. A total of 50 cycles were measured, and the curves from cycles 1, 5, 10, 20, 30, 40, and 50 were selected for comparison. The current increased with cycle number, from 0.0022 A in the first cycle to 0.0045 A in the 50th cycle. The authors attributed this result to the contribution of the nanocomposite to hydrogen evolution and to the progressive enhancement of its electrocatalytic activity during repeated cycling. This activity may be influenced by the state of the electrocatalyst deposited from the solution, charge-transfer resistance, the specific surface area of newly generated active sites, microstrain, surface defects, and oxygen vacancies.

Potentiostatic EIS measurements were conducted to determine the charge-transfer resistance (R_ct_). In this method, an alternating voltage is applied to the electrode and the resulting current response is recorded. EIS enables the electrochemical properties of the catalyst surface and phase boundaries to be examined and the charge-transfer behavior during electrocatalytic water splitting to be evaluated. The electrical equivalent circuit describing processes at the electrode–electrolyte interface comprises the solution resistance (R_s_), charge-transfer resistance (R_ct_), Warburg element (W), double-layer capacitance (Cdl), and constant-phase element (CPE).

**Figure 18:**
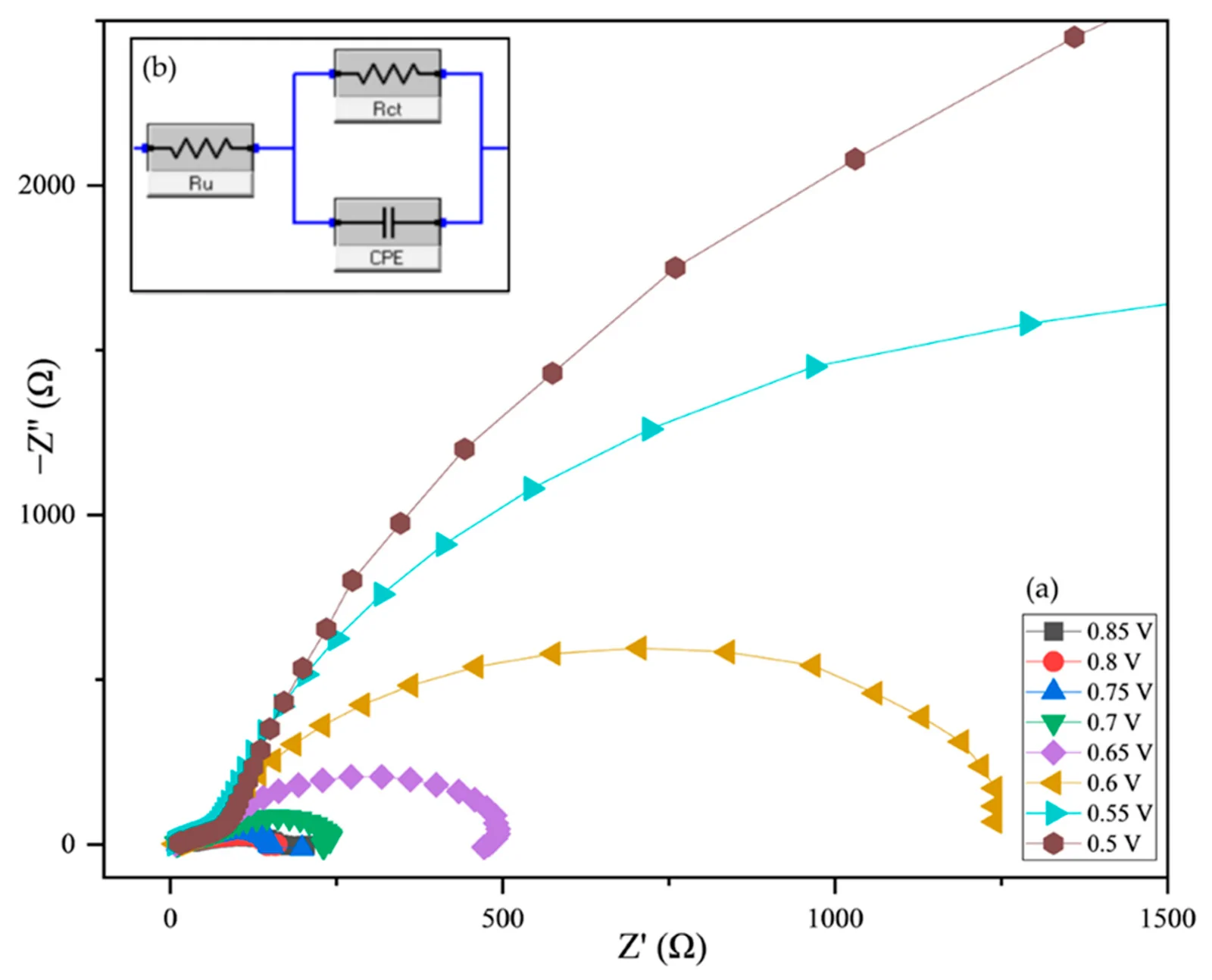
(**a**) Nyquist plot and (**b**) electrical equivalent circuit for La_0.9_MnO_3_–LaFeO_3_.

The impedance spectra of the La_0.9_MnO_3_–LaFeO_3_ nanocomposite were recorded in 0.5 M H_2_SO_4_ at different overpotentials. The data were analyzed using the simplified equivalent circuit shown in the inset of Figure 18, and the fitted results are represented by solid lines. The Nyquist plots showed that the material impedance decreased as the applied voltage increased from 0.5 to 0.85 V. At higher voltages, the contribution of the resistive component became increasingly dominant in the high-frequency region. The authors attributed this feature to a voltage-induced phase transition in the material.

Thus, the LSV results demonstrated that the electrocatalytic activity of the nanocomposite increased with cycle number, whereas EIS analysis characterized the voltage-dependent impedance response and processes occurring at the electrode–electrolyte interface [213].

## 4. Applications of Rare-Earth Orthoferrites (RFeO_3_)

**Figure 19:**
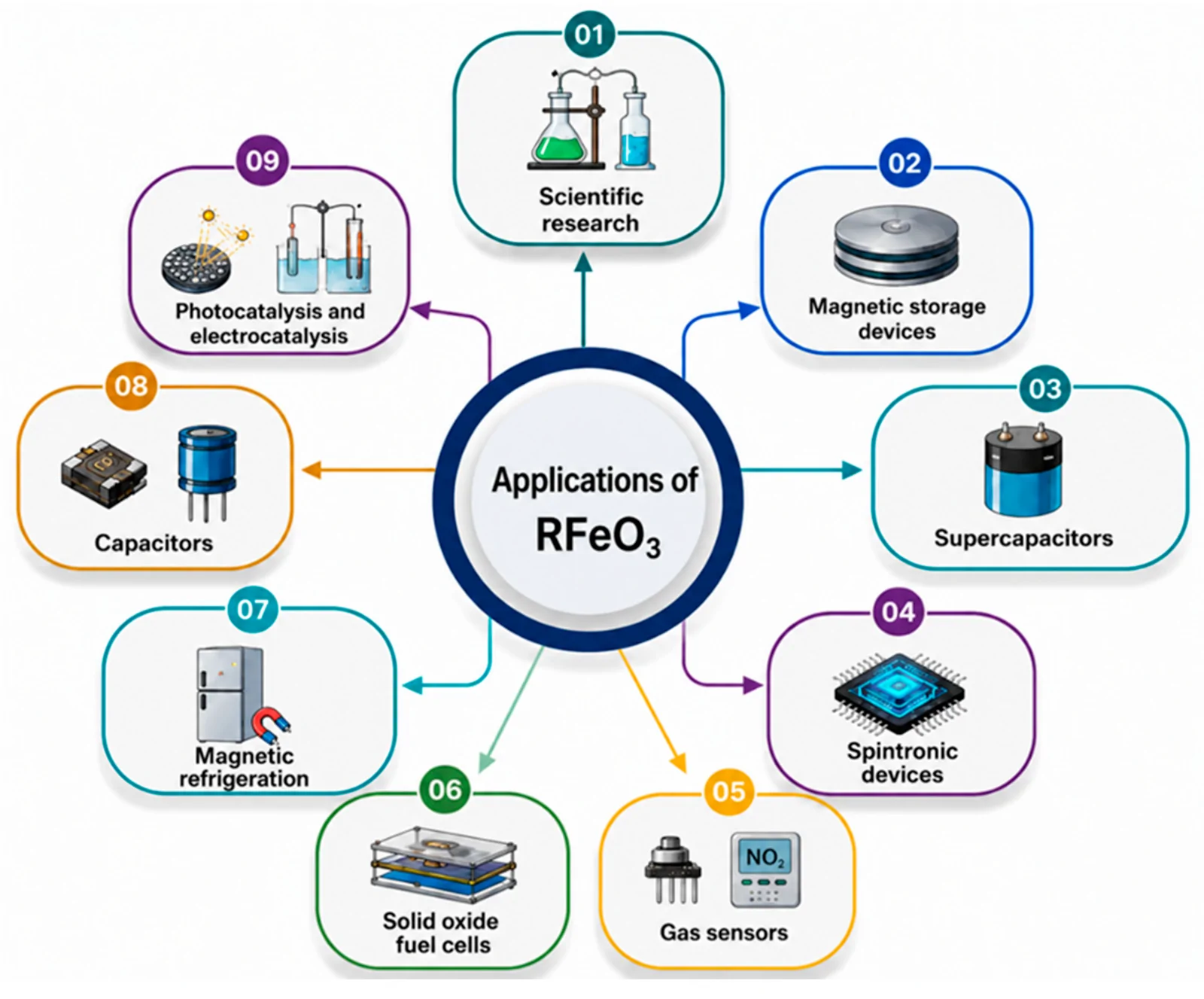
Applications of rare-earth-based orthoferrite materials.

The practical value of RFeO_3_ orthoferrites is determined not by the superiority of any single property, but by the integration of magnetic, electrical, dielectric, optical, and catalytic responses within a single structure [69]. The principal application areas are summarized in Figure 19 and discussed below in terms of the correspondence between material properties and device requirements.

### 4.1. Magnetic Refrigeration Technology

The growing need for environmentally benign and energy-efficient cooling technologies has stimulated interest in magnetic refrigeration based on the magnetocaloric effect (MCE) as an alternative to conventional vapor-compression systems. RFeO_3_ orthoferrites exhibit a distinctive combination of spin-switching, multiferroic, magneto-optical, and magnetocaloric properties.

GdFeO_3_ exhibited a magnetic entropy change of 52.5 J kg^−1^ K^−1^ under a field change of 0–9 T, demonstrating considerable potential for magnetic refrigeration below 40 K [214]. For RFeO_3_ (R = Dy, Ho, and Er) compounds, the maximum magnetic entropy changes (ΔS_m_) at 10 K under 50 kOe were 11.4, 9.8, and 7.4 J kg^−1^ K^−1^, respectively [107]. Al-doped GdFeO_3_ [215], Mn-doped TmFeO_3_ [205], and TbFeO_3_ single crystals [216] also exhibit favorable magnetocaloric characteristics. A refrigerant capacity of 497.36 J kg^−1^ was determined for TbFeO_3_ under a field of 70 kOe [217]. Overall, the tunability of orthoferrite magnetocaloric properties through elemental doping makes these compounds promising materials for low-temperature magnetic refrigeration.

### 4.2. Supercapacitors

The growing demand for efficient energy-storage and conversion systems requires the development of advanced electrode materials with high electrochemical performance. Orthoferrites are therefore being considered as promising electrode materials for next-generation supercapacitors [122]. Their electrochemical properties can be substantially improved through doping, control of synthesis conditions, and optimization of particle size.

Co-doped LaFeO_3_ containing Nd and Mn (LNFM) exhibited a specific capacitance of 158 F g^−1^ at 50 mV s^−1^, whereas a 1:1 nanocomposite of LNFM and N-doped graphene oxide (NGO) reached 1060 F g^−1^ [122]. Nd-doped SmFeO_3_ delivered 1455.9 F g^−1^ at 1 A g^−1^, approximately twice the value obtained for pristine SmFeO_3_ (746.3 F g^−1^) [88]. La_0_._7_Sr_0_._3_FeO_3_ nanofibers exhibited a capacitance of 523.2 F g^−1^ at 1 A g^−1^ in 1 M Na_2_SO_4_ [218]. Co-doped SmFeO_3_ [219] and nanoporous DyFeO_3_ electrodes [220] are likewise promising for energy-storage systems because of their high stability and electrochemical performance.

### 4.3. Spintronic and Magnetic-Switching Devices

The advancement of modern technologies, particularly next-generation spintronic devices, requires functional materials with unusual physical properties. In this context, antiferromagnetic orthoferrites (RFeO_3_, where R = La–Lu or Y) have been extensively investigated as promising materials for magnetic information processing. Their functional capabilities are associated with thermally controlled spin-reorientation transitions (SRTs) [178,221,222], magnetic-field- or temperature-induced spin switching (SSW) [64,221,222,223,224,225], compensation temperatures (T\mathrm{com}) [62,63,107], spin dynamics induced by terahertz pulses [225], and rapid spin control through optical and thermal excitation [66,226].

Li et al. [227] demonstrated that PrFeO_3_ is highly sensitive to weak magnetic fields at room temperature and proposed it as a promising material for spintronic devices operating under low magnetic fields. Kumari et al. [228] highlighted the potential of PrFeO_3_ and NdFeO_3_ for spintronic applications and ultraviolet (UV) absorption. Furthermore, doping at the R or Fe sites enables targeted control over the spin properties and magnetic anisotropy of orthoferrites. For example, a spin-reorientation transition was observed near room temperature in Sm_0_._7_Tm_0_._3_FeO_3_ [113], while investigation of a Sm_0_._5_Y_0_._5_FeO_3_ single crystal demonstrated its potential for ultrafast spintronic devices and optical magnetic-memory systems operating near room temperature [63].

### 4.4. Gas Sensors

Toxic gases emitted by industrial processes, vehicle exhaust, and fossil-fuel combustion pose serious risks to human health and the environment. The development of sensing materials capable of reliable gas detection is therefore essential. RFeO_3_ orthoferrites are regarded as promising gas-sensing materials because of their high sensitivity, selectivity, rapid response, and stability at elevated temperatures. Their distorted orthorhombic crystal structure and the presence of mixed Fe^2+^/Fe^3+^ valence states enhance gas-detection performance [123,137,229].

LaFeO_3_ exhibits high sensitivity toward nitrogen oxides [176], while Sr doping substantially improves its sensitivity and selectivity toward nitrogen oxide species [179]. SmFeO_3_ is capable of detecting ozone at sub-ppm concentrations and operates effectively even in the presence of volatile organic compounds (VOCs) [230]. In addition, Sm_0.95_Ho_0.05_FeO_3_ nanofibers exhibited a high response of 10.20 toward 5 ppm glycol [231]. In particular, doped one-dimensional RFeO_3_ nanofibers prepared by electrospinning exhibit favorable gas-sensing characteristics.

### 4.5. Solid Oxide Fuel Cells (SOFCs)

Solid oxide fuel cells (SOFCs) are environmentally benign and efficient electrochemical devices that directly convert the chemical energy of fuels into electricity. Their principal advantages include high energy efficiency, fuel flexibility, and the potential for additional utilization of the generated heat. In symmetrical solid oxide fuel cells (SSOFCs), the use of the same electrode material for both the anode and cathode simplifies the cell architecture and mitigates degradation processes such as carbon deposition and sulfur poisoning. Electrode materials for such systems must maintain high electrical conductivity and physicochemical stability under both reducing and oxidizing conditions [231].

In view of these requirements, rare-earth-based orthoferrites are being investigated as electrode and separator materials for SOFC systems. Ce-doped SmFeO_3_ has been considered as an electrode material for symmetrical SOFCs [231], whereas La_0.6_Sr_0.4_CoᵧFe_1−_ᵧO_3_ (y = 0.1–0.3) exhibited suitable characteristics as a cathode material [232]. The high electrical conductivity, thermal stability, and favorable dielectric properties of doped LaFeO_3_-based materials support their application in SOFC systems [83].

### 4.6. Magnetic Storage Devices and Information Processing

The high magnetic-ordering temperatures and multifunctional properties of orthoferrites make them promising materials for next-generation magnetic-memory devices operating at room temperature [54,223]. Magnetization reversal and controllable switching between magnetic states are key requirements for information writing, storage, and processing technologies.

NdFeO_3_ is promising for memory-based electronic devices because of its suitable band gap and pronounced magnetodielectric (MD) effect [75]. Light-induced inertial spin switching in HoFeO_3_ provides opportunities for ultrafast information-writing technologies [58]. For La_0.8_Sr_0.2_FeO_3_, saturation magnetization, remanent magnetization, and coercivity values of 8.57 emu g^−1^, 1.075 emu g^−1^, and 1994 Oe, respectively, have been reported [114]. A coercivity of approximately 748.82 Oe was also observed in Sr-doped LaFeO_3_ single crystals [101], indicating their potential for magnetic information-storage devices.

### 4.7. Capacitors

Materials with very high dielectric permittivity (ε′ ≥ 10^3^) can reduce the dimensions of capacitive elements and thereby facilitate the miniaturization of modern electronic devices [49]. Effective dielectric materials must also provide frequency stability and low dielectric loss over a broad frequency range [78].

Because of its high dielectric permittivity, LaFeO_3_ is a promising material for multilayer capacitors and resonators [81]. Ni-doped GdFeO_3_ exhibits ε′ values of approximately 10^3^–10^4^ at room temperature, demonstrating its suitability for capacitors and compact microwave resonators [232]. Co-doped LaFeO_3_ co-modified with Dy^3+^ and Ti^4+^ ions exhibits high dielectric permittivity and a low loss tangent [95]. Orthoferrites that combine high dielectric permittivity with low dielectric loss are therefore regarded as effective materials for microelectronic energy-storage devices.

### 4.8. Catalytic Applications

Organic pollutants discharged by the agricultural, textile, petrochemical, dye, cosmetics, and pharmaceutical industries pose substantial risks to human health and aquatic ecosystems. Owing to their narrow band gaps, efficient solar-light absorption, thermal stability, low toxicity, and structural flexibility, rare-earth orthoferrites have attracted interest as heterogeneous photo-Fenton-like catalysts for wastewater treatment [76,115].

Under solar irradiation, Sm_0.5_Pr_0.5_FeO_3_ exhibits high photocatalytic activity through a coupled mechanism involving semiconductor oxidation and Fenton-like reactions [115]. p-Type LaFeO_3_, with a band gap of approximately 2–2.6 eV, has been widely investigated for visible-light photocatalysis [76]. Ce incorporation enhances its visible-light absorption, whereas Cu doping improves the degradation efficiency of organic pollutants [85,86]. In addition, GdFeO_3_ and Cu-doped GdFeO_3_ exhibit high stability and activity toward the oxygen evolution reaction (OER), making them promising electrocatalysts for sustainable hydrogen production [231].

## 5. Conclusions, Key Challenges, and Future Research Directions

### 5.1. Conclusions

Rare-earth-based RFeO_3_ orthoferrites and their modified derivatives are distinguished by their ability to integrate magnetic, electrical, dielectric, optical, and catalytic properties within a single material. The literature reviewed demonstrates that their functional characteristics are governed not only by chemical composition but also by the combined effects of synthesis conditions, crystal-lattice geometry, defect structure, particle size, and interfacial interactions. Accordingly, the integrated consideration of the “synthesis–structure–morphology–property–function” relationship is essential for the rational design of orthoferrite materials.

Comparison of synthesis methods demonstrates that no universal route is equally effective for all applications. Although solid-state reactions are suitable for producing bulk ceramics and facilitating scale-up, high-temperature processing can promote particle growth and agglomeration while limiting the specific surface area. Sol–gel processing, auto-combustion, coprecipitation, and hydrothermal synthesis offer greater flexibility in controlling chemical homogeneity, particle size, and morphology; however, their outcomes depend strongly on precursor composition, solution parameters, and subsequent thermal treatment. Methods for producing single crystals, thin films, and one-dimensional structures should be selected for specific device-oriented applications. Therefore, the principal criterion for selecting a synthesis route should not be the lowest processing temperature or the smallest attainable particle size, but rather the compatibility of the resulting structure with the requirements of the intended application.

A- and B-site cation substitution, co-doping, and composite formation are complementary strategies for tailoring the properties of orthoferrites. These modifications affect FeO_6_ octahedral tilting, Fe–O–Fe bond geometry, charge-compensation mechanisms, and interfacial transport. Consequently, they alter magnetic exchange interactions, anisotropy, dielectric relaxation, electrical conductivity, and surface reactivity. Nevertheless, the observed functional enhancement cannot be adequately explained solely by the identity of the dopant ion or the presence of oxygen vacancies. Concurrent changes in phase composition, grain size, and interfacial regions must also be considered.

The application potential of orthoferrites is evident from the reported findings in magnetic refrigeration, spintronics, magnetic memory, sensors, capacitors, supercapacitors, solid oxide fuel cells, and catalytic systems. However, a high value of an individual property does not fully characterize device performance. High dielectric permittivity must be accompanied by low dielectric loss, catalytic activity by operational stability, and magnetic response by suitable operating temperatures and control conditions. From this perspective, further development of RFeO_3_ materials requires a transition from maximizing isolated performance indicators toward integrated optimization that accounts for their interdependence and application-specific constraints.

### 5.2. Key Challenges

Comparability of research findings. Synthesis protocols, sample-preparation procedures, and measurement conditions vary substantially across the literature. Failure to distinguish between particle size and crystallite size, together with incomplete reporting of density, porosity, and specific surface area, complicates the evaluation of structural differences. Direct comparison of functional parameters measured under different magnetic fields, temperatures, frequencies, electrolyte compositions, or active-material loadings may lead to unjustified conclusions.

Identification of defect structures and charge-compensation mechanisms. Oxygen vacancies and mixed-valence states are frequently invoked to explain electrical and catalytic properties, although their concentrations and spatial distributions are not directly determined in all studies. Extrapolating surface-sensitive spectroscopic features to the bulk material or assigning an individual O 1s spectral component exclusively to oxygen vacancies requires caution. It is essential to distinguish the contributions of surface hydroxyl groups, adsorbed species, and other defects.

Separation of intrinsic and extrinsic contributions. High dielectric permittivity may be influenced by grain-boundary polarization and electrode effects, whereas magnetic responses may be affected by secondary phases and domain structures. In composites, enhanced properties may arise not only from interfacial interactions but also from the intrinsic contributions of the constituent phases. Attributing an observed change to a single mechanism without separating these contributions limits the rational design of materials.

Correlation between functional parameters and underlying mechanisms. A decrease in band-gap energy does not necessarily imply improved photocatalytic performance; charge separation, recombination, and surface reactions must also be evaluated. Similarly, an increase in electrocatalytic current or a decrease in impedance does not, by itself, demonstrate efficient product formation. Likewise, enhanced magnetic anisotropy or coercivity is not equally advantageous for every spintronic or magnetic-memory device.

Stability and technological implementation. A substantial proportion of the reviewed studies focuses primarily on the initial characteristics of the materials. Long-term cycling, exposure to chemical environments, thermal transitions, and surface reconstruction under operating conditions have not been sufficiently compared. Synthesis reproducibility, energy consumption, the availability of rare-earth components, and retention of material properties during scale-up require separate evaluation before practical implementation.

### 5.3. Future Research Directions

Controlled comparative studies. For a given initial composition, the synthesis method, calcination temperature, or doping level should be varied independently while all other parameters are maintained constant. Such an approach would enable compositional, dimensional, and defect-related effects to be distinguished. Comprehensive reporting of measurement conditions, replicate experiments, and associated uncertainties would provide a reliable basis for comparing results across different studies.

Quantitative and spatially resolved investigation of defect chemistry. Complementary characterization techniques should be employed to determine oxygen stoichiometry, cation oxidation states, and defect distributions at the surface and within the bulk. Combining XPS data with bulk structural and chemical analyses would help clarify charge-compensation mechanisms. In situ and operando measurements under realistic operating conditions are essential for determining the extent to which the initial structure is retained during catalytic or electrical operation.

Targeted control of phase boundaries and hierarchical structures. In composite materials, greater emphasis should be placed on controlling phase ratios, interfacial contact area, grain-boundary structure, and interfacial defects. This strategy could enable the coordinated regulation of charge transport, magnetic exchange interactions, and photoinduced charge separation. To substantiate interfacial effects, composites should be compared with their individual constituent phases and with physical mixtures containing the same phase ratios.

Development of application-specific evaluation criteria. In catalytic systems, activity should be assessed together with product yield, selectivity, Faradaic efficiency, reusability, and component leaching. For supercapacitors, electrode-level parameters should be complemented by the energy and power characteristics of complete devices. Sensor sensitivity should be evaluated together with selectivity, response time, and long-term signal stability. Magnetic and dielectric materials should be assessed under realistic operating temperatures, frequencies, field strengths, and loss requirements.

Integration of theoretical modeling and experiment. Electronic-structure calculations, defect-formation modeling, and analysis of magnetic interactions should be directed not only toward interpreting experimental findings but also toward identifying promising new compositions. The establishment of quality-controlled datasets containing complete measurement conditions would enable data-driven optimization of composition and synthesis parameters. Predictions generated by such models should subsequently be validated through independent experiments.

Development of reproducible and resource-efficient technologies. Low-temperature synthesis, reduced processing times, and the use of readily available components should be combined with the preservation of material stability and functional performance. During the transition from laboratory-scale samples to device prototypes, batch-to-batch consistency, technological compatibility, environmental safety, and overall production costs must be evaluated.

The coordinated implementation of these research directions would facilitate the transition from empirical screening of RFeO_3_ orthoferrites to rational design based on their structure and operating mechanisms. The principal objective is not merely to achieve the highest value of an individual performance indicator, but to integrate the required properties within a stable, reproducible, and technologically viable material.

## Figures and Tables

**Table 1:** Comparative characteristics of synthesis methods for RFeO_3_-type perovskite orthoferrites.

Synthesis Method	Key Synthesis Parameters	Particle/Crystallite Size	Advantages	Limitations	References
Solid-state reaction	Calcination/sintering temperature: 900–1500 °C	10–15 nm to 5.6 μm; 27–88 nm reported in other studies	Technologically straightforward; readily available starting reagents; relatively low cost; suitable for the large-scale production of mixed oxides	Requires high temperatures and prolonged heat treatment; prone to agglomeration; precise control of particle size is difficult; stoichiometric inhomogeneity and secondary-phase formation may occur	[49,51,52,54,55,57,59,60,95,96,97,98,99,147]
Optical floating-zone method	Single crystals are grown from a high-temperature molten zone	Intended for single-crystal growth	Produces high-quality crystals; crucible-free and flux-free process; low risk of contamination by foreign impurities; relatively high crystal growth rate	Requires specialized equipment; precise control of molten-zone stability and temperature is essential; dislocations, cracks, and gas bubbles may form	[61,62,63,64,65,66,100,101]
Sol–gel method	High degree of precursor mixing in the liquid phase; phase formation occurs at a lower temperature than in the solid-state method	SmFeO_3_: approximately 65 nm; LnFeO_3_: 40–80 nm; doped systems: 20–252 nm	High chemical homogeneity and phase purity; enables the production of nanoscale materials; particle size, structure, and physical properties can be tailored	Involves multiple processing stages; gelation, drying, and calcination conditions require careful control; strongly dependent on precursor and solvent properties	[67,68,69,70,71,72,73,74,75,102,103,104,105,106,107,108]
Sol–gel auto-combustion	Sol → xerogel → self-combustion; glycine, urea, or citric acid is used as the organic fuel	15–365 nm; 15–50 nm in many studies	Technologically simple and cost-effective; high chemical homogeneity, phase purity, and crystallinity; enables the production of nanoparticles with a high specific surface area	The outcome depends strongly on fuel type and combustion conditions; grain growth and agglomeration may occur during subsequent calcination	[76,79,109,110,111,112,113,114,115,116]
Coprecipitation method	Simultaneous precipitation of metal cations from a single solution, followed by calcination	Typically 18.2–77 nm; HoFeO_3_: 25–30 nm; 0.2–2 μm in certain cases	Mixing of components at the atomic scale; high chemical and phase homogeneity; relatively low calcination temperature; suitable for producing fine particles	Requires precise control of pH, concentration, temperature, and stirring rate; component solubilities must be compatible; prolonged calcination increases processing time and energy consumption	[80,81,82,83,84,85,117,118,119,120,121]
Hydrothermal/solvothermal method	Conducted in an autoclave under controlled temperature and pressure; requires a relatively low post-synthesis heat-treatment temperature	30–70 nm and 31–39 nm; 260–427 nm in some systems	Produces materials with high phase purity and crystallinity; relatively narrow particle-size distribution; enables morphological control and the formation of nanospheres, nanoflowers, nanoplates, and nanorods	Requires a specialized autoclave; high pressure imposes stringent safety requirements; temperature, pH, precursor concentration, and reaction time must be precisely controlled	[86,87,88,89,90,102,103,122,123]

**Table 2:** Crystal-lattice parameters of the LaFeO_3_ phase determined by Rietveld analysis.

Sample	Perovskite Phase, %	Crystallite Size ^a^, nm	Microstrain ^a^, %	a, Å	b, Å	c, Å
8LFO20-0	97	100.55 (4)	0.030 (7)	5.5638 (2)	7.8546 (2)	5.5558 (2)
8LFO20-1	97	91.08 (3)	0.051 (5)	5.5645 (2)	7.8553 (3)	5.5562 (2)
8LFO20-2	97	101.53 (4)	0.075 (3)	5.5643 (2)	7.8549 (3)	5.5560 (2)
8LFO20-5	98	91.14 (4)	0.108 (3)	5.5622 (4)	7.8553 (5)	5.5561 (3)

Note: ^a^ The parameters were calculated by profile refinement using the Rietveld method in HighScore Plus software. The statistical errors given in parentheses correspond to the estimated standard deviations (ESDs) derived from the least-squares covariance matrix.

**Table 3:** Dielectric properties and key characteristics of doped and undoped orthoferrites.

Material and Synthesis Method	Dielectric Permittivity (ε′)	Dielectric Loss (tan δ)	Key Characteristics	Ref.
SmFeO_3_: solid-state (SFO-s), nanocombustion (SFO-n)	At room temperature: ≈45 (SFO-s), >1000 (SFO-n)	SFO-s: stable up to 450 K; SFO-n: increases with temperature	SFO-n exhibits lower resistance (≈10^6^ Ω); the high ε′ is associated with microstructural features and synthesis conditions	[181]
LuFe_1−x_Ir_x_O_3_, x = 0, 0.05, 0.10; solid-state	ε′ is higher for LFO	Lowest at 5 mol.% Ir	Ir incorporation reduces the Fe^2+^ fraction and decreases ε′ and electrical conductivity as a result of structural distortion	[189]
Dy^3+^/Ti^4+^–LaFeO_3_, solid-state	Increases upon doping	Decreases	Electrical resistivity increases while conductivity decreases; a ferroelectric–paraelectric transition occurs at ≈670 K	[95]
LaFeO_3_, solid-state	Very high	Minimal	Multiferroic behavior and non-Debye dielectric response are observed	[182]
LuFeO_3_ and Lu_0.9_Co_0.1_Fe_0.9_Ti_0.1_O_3_, sol–gel	LFO ≈261; LCFTO ≈1053	Very low	Co-doping enhances the ferroelectric response by 2.36-fold and the magnetocapacitance by ≈4.3-fold	[108]
Sm_0.5_R_0.5_FeO_3_, R = Gd, Nd, Pr; auto-combustion	At 42 Hz: Sm—2640; Gd—2330; Nd—2424; Pr—2830	0.69; 0.74; 0.57; 0.52	ε′ decreases with increasing frequency and becomes nearly frequency-independent at high frequencies	[115]
SmFe_1−x_Mn_x_O_3_, x = 0.1–0.5; sol–gel	Increases with Mn content	Decreases	Mn incorporation enhances electrical conductivity	[190]
SmFe_1−x_Cr_x_O_3_, x = 0.1–0.3; sol–gel	2532–1172 at 1 kHz; SmFeO_3_—2914	Increases with Cr content	The decrease in ε′ is associated with IBLC effects and dipolar polarization; Cr^2+^ promotes grain growth	[190]
TbFeO_3_, solid-state	≈48,800 at 100 Hz and 160 °C	Relaxation maximum above 75 °C	Multiple valence states of Tb ions and relaxation processes are observed	[50]
LaFeO_3_ and La_0.85_Gd_0.15_Fe_0.9_Ti_0.1_O_3_, solid-state	Lower for LGFTO	Lower for LGFTO	Doping improves resistivity and P–E characteristics	[51]
SmFeO_3_, Sm_0.9_Bi_0.1_FeO_3_, Sm_0.9_Bi_0.1_Fe_0.9_Mn_0.1_O_3_, solid-state	≈10^3^–10^4^	Low at high frequency	ε′(SBFMO) > ε′(SFO), but ε′(SBFMO) < ε′(SBFO)	[55]
Ce_0.5_Sm_0.5_FeO_3_, solid-state	High	Minimal at room temperature	tan δ is associated with Fe^2+^–Fe^3+^ double exchange and Fe^3+^ spin configurations	[56]
PrFe_1−x_Mn_x_O_3_, x = 0–0.5; solid-state	Increases with Mn content	Low at high frequency	Increasing Mn content decreases the activation energy (Eσ) and optical band-gap energy (Eg)	[57]
GdCo_x_Fe_1−x_O_3_, x = 0–0.3; solid-state	Increases with Co content, with a maximum at x = 0.2	Decreases with frequency	AC conductivity increases with frequency and reaches a maximum at x = 0.2	[59]
Ce–NdFeO_3_, sol–gel	Decreases with increasing Ce content	<1	AC conductivity increases with frequency but decreases with increasing Ce content	[73]
HoFeO_3_ and Ho_0.95_Co_0.05_Fe_0.95_Ti_0.05_O_3_, sol–gel	≈93 and ≈254	Decreases by ≈50%	Polarization with low leakage loss is observed at room temperature	[74]
La_1−x_Nd_x_FeO_3_, x = 0–0.5; sol–gel auto-combustion	≈11-fold increase upon Nd substitution	Low	At x = 0.3, both ε′ and AC conductivity increase substantially	[78]
Er_1−x_La_x_FeO_3_, x = 0–1; co-precipitation	Increases from ≈20 to ≈3600	Multiple relaxation processes	The increase in ε′ is associated with La-induced microstructural modifications	[81]
LaFeO_3_ and Ce–LaFeO_3_, microwave-assisted gel combustion	Decreases upon Ce incorporation	Decreases by ≈2 orders of magnitude	Ce incorporation induces magnetodielectric coupling and reduces leakage current	[91]
LaFeO_3_ and La_0.9_Al_0.1_Fe_0.9_Ti_0.1_O_3_, solid-state	Higher for LAFTO	Lower for LAFTO	Pmax = 0.25 μC/cm^2^; ferroelectric–paraelectric transition at ≈655 K; the structure transforms to Pna2_1_	[97]
LaFeO_3_ and La_0.9_Sr_0.1_FeO_3_, optical floating-zone	At 742 K: 2.25 × 10^5^ and 3.25 × 10^5^	Increases with temperature	The high ε′ is associated with the mobility of Fe^3+^ ions	[103]
La_1−x_Pb_x_FeO_3_, x = 0.15, 0.25; solid-state	≈10^4^ above 290 K	Multiple relaxation maxima	Electrical inhomogeneity and multiple relaxation processes are observed over 80–400 K	[191]
LaFeO_3_ and La_0.9_Er_0.1_FeO_3_, modified sol–gel	Increases from ≈48 to ≈92	≈10^−4^ at high frequency	Dielectric phase transitions occur at ≈683 and 691 K, respectively	[188]
SmFeO_3_, citric-acid method	≈10^4^ at 100 Hz and room temperature	Frequency- and temperature-dependent	The high ε′ is mainly governed by grain-boundary effects	[192]
La_1−x_Na_x_FeO_3_, x = 0–0.2; sol–gel	≈10^5^ at 100 Hz for the doped sample	Increases upon Na incorporation	Na increases grain size and ε′, but also increases tan δ	[193]
LaFeO_3_ and La_0.8_Gd_0.2_Fe_0.97_Nb_0.03_O_3_, solid-state	High at low frequency	Lower for LGFNO	High ε′ is associated with grain boundaries, dislocations, and oxygen vacancies; a phase transition occurs at ≈780 K	[194]
La_1−x_Al_x_FeO_3_, x = 0.05–0.25; solid-state	213–771	Minimum at x = 0.15	The x = 0.15 composition is promising for high-frequency applications	[195]
GdFe_1−x_Ni_x_O_3_, x = 0–0.2; solid-state	≈10^3^–10^4^	Depends on Ni content	The high apparent ε′ is mainly governed by extrinsic effects; intrinsic ε′ ≈16–20	[196]
PrFe_1−x_Cr_x_O_3_, x = 0–0.20; sol–gel	Not reported	Increases with Cr content	Dielectric loss is associated with electron tunneling transport	[197]

**Table 4:** Magnetic properties and characteristic parameters of the investigated materials.

Sample	M_s_ (emu g^−1^)	H_c_ (G)	M_r_ (emu g^−1^)	S	K	μB
SmF	0.796	505.8	0.209	0.262	419.39	0.0362
NiF/SmF	36.526	172.9	10.87	0.298	6578.5	3.1953
CoF/SmF	41.584	1941	19.747	0.475	84.078	3.6396
ZnF/SmF	15.3	63.188	2.616	0.179	1007.1	1.3568
CdF/SmF	22.571	114.6	3.672	0.163	2694.4	2.1918

## Data Availability

No new data were created or analyzed in this study. Data sharing is not applicable to this article.
